# Deceptive bias measurement in deep learning: Assessing shortcut reliance in TCGA cancer models

**DOI:** 10.1371/journal.pdig.0001165

**Published:** 2026-09-03

**Authors:** Farnaz Kheiri, Shahryar Rahnamayan, Masoud Makrehchi

**Affiliations:** 1 Department of Electrical, Computer and Software Engineering, Ontario Tech University, Oshawa, Ontario, Canada; 2 Department of Engineering, Brock University, St. Catharines, Ontario, Canada; National University of Singapore, SINGAPORE

## Abstract

Machine learning bias is a persistent challenge because it can create unfair outcomes, limit generalization, and reduce trust in real-world applications. A key source of this problem is shortcut learning, where models exploit signals linked to sensitive attributes, such as the data source or collection site, instead of relying on task-relevant features. To address this, we propose the Deceptive Signal metric, a novel quantitative measure designed to assess the extent of a model’s reliance on hidden shortcuts during the learning process. This metric is derived via the Deceptive Bias Detection pipeline, which isolates shortcut dependence by contrasting the model’s behavior under two controlled conditions: (1) Full Exclusion, where a sensitive subgroup is completely removed from training; and (2) Partial Exclusion, where the model has limited access to specific classes within that subgroup. By calculating the behavioral shift between these settings, the Deceptive Signal metric provides a quantitative estimate of the model’s susceptibility to learning task-irrelevant patterns. In experiments with the TCGA histopathology dataset, our metric successfully quantified substantial dependencies on center-specific artifacts in models trained for cancer classification.

## Author summary

Deep learning models are becoming powerful tools in healthcare, but they often suffer from a critical vulnerability: they can get the right answer for the wrong reason. In medical imaging, an AI might correctly identify a tumor not by analyzing the tissue, but by recognizing irrelevant digital markers unique to the specific hospital or scanner that produced the image. This phenomenon, known as shortcut learning, makes AI systems appear accurate at first glance while potentially limiting reliability in real-world patient care settings.To solve this, our research moves beyond simple accuracy checks and introduces a specific quantitative metric for shortcut learning. We developed a testing framework that forces the model into controlled training scenarios, deliberately withholding specific shortcut information to see how the model reacts. By mathematically comparing the model’s behavior across these scenarios, we calculate a quantitative score that indicates the magnitude of the model’s dependence on irrelevant patterns. This metric allows us to put a concrete number on the model’s trustworthiness, helping assess whether predictions are more strongly driven by task-relevant biological features rather than background artifacts.

## 1. Introduction

In recent years, the increasing integration of intelligent systems into various aspects of daily life has underscored the critical need to develop models that are both fair and unbiased. Bias in machine learning poses serious risks; it can produce discriminatory outcomes, compromise model generalization to unseen data, and raise significant ethical and societal concerns [1–3]. To ensure fairness, models must rely on task-relevant features rather than exploiting shortcuts associated with sensitive factors such as gender or data origin (e.g., specific data collection sites or institutions). As the foundation of any predictive system, training data play an important role in shaping model outputs. Therefore, the presence of easy-to-learn but task-irrelevant patterns associated with sensitive attributes may severely undermine model trustworthiness, particularly when deployed in real-world settings or evaluated on external samples containing sensitive attributes not represented during training [4,5].

One critical step during the training process is to assess whether a model is truly learning from task-relevant patterns or if its predictions are influenced by task-irrelevant shortcuts. These shortcuts often stem from spurious correlations or hidden signatures associated with sensitive attributes, such as demographic features or dataset origins [6,7]. Evaluating how model behavior changes in the presence or absence of these sensitive attributes is essential for identifying potential sources of bias. Such investigations help ensure that the model’s decision-making process remains aligned with the intended task rather than being skewed by irrelevant or discriminatory patterns embedded in the training data [8,9]. A notable example of this issue arises in medical imaging datasets [10], such as TCGA, where samples are collected from multiple data centers. These centers may differ in scanning devices, staining protocols, or other characteristics, unintentionally embedding identifiable patterns into the images. Consequently, models may learn to associate such center-specific artifacts with the target labels (e.g., cancer type), leading to biased predictions that do not generalize well outside the training distribution [11,12].

Many existing bias investigation methods focus on disparities in prediction outcomes across different sensitive groups, such as the positive prediction rate [13]. However, they often overlook the underlying learning behavior during training. These approaches tend to assess bias after the model is trained, by measuring how its predictions vary across subgroups [14]. However, such outcome-based evaluations may fail to detect whether the model has relied on task-irrelevant features embedded within specific subgroups. To better assess fairness and generalization, it is crucial to go beyond surface-level performance metrics and analyze the training process itself by examining whether the model is learning representations that generalize to the task or simply memorizing subgroup-specific artifacts.

To evaluate the reliability of deep learning models in multi-center clinical data, this study addresses three primary research questions:

- **RQ1:** To what extent do deep models rely on site-specific artifacts rather than morphological features for classification in multi-center datasets?
- **RQ2:** Can a controlled exclusion-inclusion training procedure effectively isolate and quantify these hidden shortcuts through behavioral shifts?
- **RQ3:** Does the proposed Deceptive Signal (*S*) provide a stable, architecture-agnostic metric for auditing bias across diverse clinical settings?

## 2. Related work

To contextualize the challenges of fairness and generalization, we review prior studies on bias detection and quantification. Here, we examine how recent research has attempted to assess these biases. We categorize existing methodologies into two primary streams: techniques that investigate hidden biases and spurious correlations within model representations, and outcome-based methods that evaluate fairness through disparities in predictive performance across sensitive subgroups.

### 2.1. Shortcut learning and hidden bias in representations

Here, we focus on studies demonstrating how deep models may learn spurious correlations or shortcuts (e.g., background patterns, color artifacts, dataset source patterns) and employ these signatures to fulfill the target task [15]. Geirhos et al. [5] demonstrated that models trained on a modified version of ImageNet, in which textures were removed while object shapes were preserved, performed significantly worse than models trained on the original ImageNet. This reveals that the models relied heavily on texture-based cues during training.

Saliency-based methods are a popular approach widely used in prior studies to identify the regions or features within an input that most influence a model’s predictions. Techniques such as Grad-CAM [16–18] and saliency maps [19–21] aim to enhance the interpretability of model outputs by identifying which parts of the input data have the greatest impact on the model’s decisions during training or inference. Studies [22–28] applied saliency map techniques to medical images to investigate whether diagnostic decisions were based on task-relevant features or influenced by spurious, non-diagnostic patterns. Results reveal that in many cases, deep models tend to focus on artifacts such as image borders, text annotations, or scanner-specific features, rather than the actual pathological regions.

Another widely used methodology is based on counterfactual scenarios [29], which aim to assess the fairness of model outputs by examining their sensitivity to changes in sensitive attributes. These methods involve modifying input features, typically by altering or removing sensitive variables such as gender or race [30–32], while keeping the target class constant. If the model’s prediction changes significantly after such a modification, it suggests that the model may be relying on task-irrelevant or sensitive features, rather than learning from the core features relevant to the task.

Autoencoders can help uncover shortcuts by learning a compact version of an image and reconstructing it to see what information and patterns are truly learned by the model. If the reconstruction removes irrelevant details (like scanner artifacts) and the main model’s accuracy drops, it suggests the model was relying on those irrelevant details rather than the real features [17,33,34].

### 2.2. Outcome-based fairness evaluation

Another line of research in this section focuses on evaluating the impact of sensitive attributes on model outcomes using fairness metrics, typically by measuring disparities in predictive performance across different sensitive subgroups. These outcome-based fairness metrics operate under the assumption that a fair model should yield comparable performance or decision rates across all groups, regardless of the sensitive attributes to which they belong. The method proposed in [35] applies the definition of group fairness metrics [14] to quantify the effect of encoded shortcuts on model outcomes. They measure disparities using metrics such as the True Positive Rate or the Equal Opportunity metric [36] across continuous sensitive attributes. The study in [37] employs Worst-Group Accuracy (WGA) to assess shortcut reliance by defining groups as combinations of labels and sensitive attributes. Lower WGA in fairer models suggests that removing shortcut signals can reduce performance on disadvantaged subgroups. Further research [38] applied the Equalized Odds metric to assign weights to balance class distributions within each demographic group to ensure comparable true positive and false positive rates. Studies [27,39] address the shortcut learning issue by reducing disparities in metrics such as the Area Under the Curve, Binary Cross-Entropy, Expected Calibration Error, and Precision through a model and task-agnostic image augmentation strategy.

In this work, we address this gap by proposing a novel measurement metric designed to quantify the extent to which a model relies on task-irrelevant patterns associated with sensitive attributes. To derive this metric, we introduce the Deceptive Pipeline, a structured methodology that manipulates the training data to examine how these patterns influence model decisions and lead to biased behavior.

It is important to note that we do not propose the Deceptive Signal (*S*) as a replacement for established multi-faceted auditing protocols, such as dataset representation analysis, saliency auditing, or subgroup performance metrics. Instead, we view our metric as a complementary test within a bias verification protocol. While visual methods provide qualitative evidence of where a model is looking, the Deceptive Signal adds a layer of mathematical precision by specifically quantifying the impact of shortcut learning on model behavior.

We validate our approach using the TCGA dataset, which comprises samples collected from various medical centers. Prior studies [8,12] have demonstrated that deep models trained on TCGA often capture data center-specific artifacts and leverage them as hidden shortcuts for cancer classification. Extending these findings, we analyze the training phase itself to assess the reliability of the learning procedure, determining whether the model is truly learning cancer-related characteristics or is driven by data center-specific signatures.

The Deceptive Pipeline facilitates this measurement by altering the presence or absence of specific sensitive attribute combinations via multiple training setups. This allows us to test whether the model learns task-relevant (cancerous) features or simply relies on task-irrelevant patterns tied to data center origin. By observing shifts in misclassification patterns across these controlled settings, we gain insight into how shortcut-related bias may emerge even before the evaluation phase. Consequently, the core contribution of this study is a quantitative metric that estimates the model’s susceptibility to learning these task-irrelevant shortcuts.

Our proposed methodology consists of three structured phases to calculate this metric. **Phase 1 (Full Shortcut Exclusion)** involves training the model without any samples from a specific sensitive subgroup to assess its performance without exposure to subgroup-specific features. **Phase 2 (Partial Shortcut Exclusion)** selectively includes samples from only one class within the sensitive subgroup, allowing us to observe how partial exposure impacts classification behavior. Finally, **Phase 3 (Deceptive Signal Measurement)** compares the misclassification patterns between the full and partial exclusion settings to compute the proposed metric, characterizing the magnitude of the model’s dependence on task-irrelevant features.

## 3. Methodology

Advanced machine learning models, including deep neural networks, may rely on unintended correlations or shortcuts present in the input data to complete assigned tasks. These shortcuts may be associated with sensitive attributes, such as gender or with irrelevant patterns, such as unique signatures specific to certain data sources that are unrelated to the task’s primary objective. When models depend on such attributes or patterns, their predictions may become less reliable under distribution shifts. This unreliability becomes more evident when input samples that lack these attributes are fed into the model; this can lead to poor performance.

To clarify this concept with a real-world example, imagine a math teacher designing exam questions for students. These questions are nearly identical to those solved during tutorial sessions, with only minor numerical modifications. In such a case, exam results would primarily reflect how well students have memorized specific solutions, rather than their understanding of the underlying concepts. The Deceptive Bias Detection method is designed to reveal such reliance by systematically modifying model inputs to remove access to the sensitive signal and then reintroducing it during testing or inference to measure the resulting behavioral shift.

### 3.1. The deceptive pipeline principle

The core hypothesis behind our Deceptive Bias Detection pipeline is that if a model relies on hidden patterns or sensitive attributes as shortcuts to generate its output, its behavior is expected to change measurably when those attributes are removed during training and then reintroduced during evaluation. Such a behavioral shift suggests that the model has encoded a dependency on those attributes. Depending on the specific use case, this hypothesis can be adapted to target specific types of bias, such as gender, source-specific artifacts, or domain-related features, by manipulating or masking the relevant attributes during different stages of training and evaluation. The proposed pipeline consists of three major phases:

#### 3.1.1. Phase 1. Full shortcut exclusion.

We first prepare a controlled training input: data carefully selected to exclude shortcuts associated with sensitive attributes or other patterns whose influence we wish to study. The goal is to ensure that the model has no access to the attribute or pattern under investigation. The model is then trained using this controlled input, and the generated outputs are evaluated for further analysis.

For example, when investigating whether data center-specific patterns influence the learning process, we exclude all data from a particular center during training and then evaluate the model’s performance on test data from that same center. This approach can also be used to evaluate aspects of model generalizability, as it reveals how well a model can perform on completely unseen subsets associated with specific attributes. Notably, the test data are balanced across both class types and sensitive groups. We maintain the same test data throughout all steps of our pipeline.

#### 3.1.2. Phase 2. Partial shortcut exclusion.

In this phase, we exclude only a portion of the training data associated with the target sensitive attribute or the shortcut pattern rather than completely removing it. The objective is to expose the model to limited cues, allowing us to observe how partial exposure affects model behavior.

Building on the previous example, instead of excluding the entire dataset from a particular center, we now exclude only a specific class from that center while keeping other classes from the same center in the training set. During testing, we evaluate the excluded class from that center to observe whether it gets misclassified as one of the included classes belonging to the target sensitive attribute.

#### 3.1.3. Phase 3. Deceptive signal measurement.

In this phase, we aim to quantify the behavioral shift resulting from the evaluations performed in Phase 1 and Phase 2. The main goal is to compare how the model’s responses or predictions change when exposed to different levels of access to the sensitive attribute or shortcut pattern.

The method of comparison may vary depending on the specific application. For example, when evaluating how cancer-related features are influenced by data center-specific patterns in medical imaging, we may use classification accuracy shifts, confusion matrix disparities, or representation analysis. The next section presents a case study and the corresponding evaluation procedures used to assess whether models trained for cancer classification rely more on task-relevant patterns or on center-specific artifacts.

### 3.2. Mathematical formalization

Let $D=\{(x_{i},y_{i}){\}}_{i=1}^{n}$ be a dataset containing samples $x_{i}$ with class labels $y_{i}$. Let $S_{k}$ denote a sensitive subgroup (e.g., data from a specific data center), and let $C_{i}$ represent a particular class label of interest.

#### 3.2.1 Phase 1. Full Exclusion.

In this phase, we train a model $f_{-S_{k}}$ using all data in *D* except those belonging to subgroup $S_{k}$, i.e., $D⧵S_{k}$.

We then evaluate $f_{-S_{k}}$ on $D_{test}^{C_{i},S_{k}}$, which is the subset of test data where the samples belong to class $C_{i}$ and originate from subgroup $S_{k}$. This setup allows us to measure how well the model generalizes to $S_{k}$ when it has not seen any data from that subgroup during training. Subsequently, we calculate $DP_{C_{i},S_{k}}^{\text{Full}}$ as the proportion of test samples from class $C_{i}$ in subgroup $S_{k}$ that are misclassified by the model $f_{-S_{k}}$.


$$\begin{array}{c} DP_{C_{i},S_{k}}^{\text{Full}}=\frac{\left|\left\{x\in D_{\text{test}}^{C_{i},S_{k}}:f_{-S_{k}}(x)\right\}\right|}{\left|D_{\text{test}}^{S_{k},C_{i}}\right|} \end{array}$$ (1)

The expression of the numerator counts how many test samples of class $C_{i}$ and subgroup $S_{k}$ were misclassified by the model $f_{-S_{k}}$, which was trained without seeing any samples from $S_{k}$. The denominator is the total number of test samples from subgroup $S_{k}$ and belonging to $C_{i}$. This ratio quantifies the model’s error rate for a specific class–subgroup pair in a completely unseen subgroup scenario.

#### 3.2.2. Phase 2. Partial exclusion.

In the second phase, we train the model $f_{-(S_{k},C_{ex})}^{\prime }$ by excluding only class $C_{ex}$ from $S_{k}$ and including other classes belonging to $S_{k}$ in the training set. We then re-test the model using the same test set to calculate the proportion of misclassified samples:


$$\begin{array}{c} DP_{C_{i},S_{k}}^{\text{Partial}}=\frac{\left|x\in D_{test}^{C_{i},S_{k}}:f_{-(S_{k},C_{ex})}^{\prime }(x)\right|}{\left|D_{test}^{S_{k},C_{i}}\right|} \end{array}$$ (2)

This equation measures how task-irrelevant features affect model performance by examining whether including partial classes causes the model to be deceived by irrelevant features embedded in those samples.

#### 3.2.3. Phase 3. Deceptive signal measurement.

In this phase, we quantify the degree to which a model relies on shortcut patterns by comparing its behavior under two training conditions: full shortcut exclusion (as in Phase 1) and partial shortcut exclusion (as in Phase 2). The key idea is to measure how much the model’s predictions shift when it is trained with limited class access to a sensitive attribute, compared to when it has no access to that attribute across any class. Essentially, we use the included classes from $S_{k}$ to determine whether the model is deceived by $S_{k}$-specific features after partial exposure.

To capture this behavioral change, we define the Deceptive Disparity Shift ($\Delta DP_{C_{i},S_{k}}$) as the difference between the misclassification rate under partial and full exclusion for each class within the sensitive subgroup $S_{k}$. Here, our objective is to compare the disparity shift of the excluded class within the training process with the included classes of $S_{k}$. This is calculated as:


$$\begin{array}{c} \Delta DP_{C,S_{k}}=DP_{C,S_{k}}^{\text{Partial}}-DP_{C,S_{k}}^{\text{Full}} \end{array}$$ (3)

If the number of misclassified samples in the partial exclusion setting is greater than in the full exclusion setting, this suggests something important. In partial exclusion, the model has seen more data from the sensitive group $S_{k}$, since only one class from $S_{k}$ was left out during training. Conversely, in full exclusion, the model has seen no data at all from $S_{k}$. So, if the model performs worse in the partial case, even with more related training data, it means the model is likely using patterns (or shortcuts) embedded in $S_{k}$ samples from the seen class to make predictions about the unseen class in the same group instead of task-relevant features. This behavior indicates that the model has learned to rely on group-specific features that are not truly relevant to the task.

To aggregate the calculated values across classes and derive the bias metric, we first compute the average disparity shift for the included classes within $S_{k}$, denoted as $I_{S_{k}}$, and treat this value as the baseline. This average is defined as:


$$\begin{array}{c} {\text{Avg}}_{I_{S_{k}}}=\frac{1}{\left|I_{S_{k}}\right|}\sum_{C_{j}\in I_{S_{k}}}\Delta DP_{C_{j},S_{k}} \end{array}$$ (4)

The deceptive signal over $S_{k}$ is then obtained by subtracting this baseline from the disparity shift of the excluded class within this sensitive group.


$$\begin{array}{c} \text{Deceptive Signal}=\Delta DP_{C_{ex},S_{k}}-{\text{Avg}}_{I_{S_{k}}} \end{array}$$ (5)

To interpret the bias metric meaningfully, it is useful to examine its behavior under extreme scenarios. Such scenarios reveal the theoretical limits of the metric and establish its lower and upper boundaries. We illustrate these limits through two contrasting cases: the *best case*, representing an unbiased model, and the *worst positive case*, reflecting strong reliance on shortcut patterns associated with the sensitive subgroup $S_{k}$.

In the given equations, both *DP*^Partial^ and *DP*^Full^ are proportions of misclassified samples, and therefore take values in the range [0, 1]. This means that each $\Delta DP_{C,S_{k}}$ lies within [−1,1]. Because each $\Delta DP_{C,S_{k}}$ can only take values between −1 and 1, the average of these values across the included classes must also fall within the same range. Therefore, ${\text{Avg}}_{I_{S_{k}}}$ is bounded by −1 and 1.

With these bounds in place, the Deceptive Signal Measurement reaches its maximum value when the disparity shift for the excluded class is as large as possible $\left(\Delta DP_{C_{ex},S_{k}}=1\right)$ and the average disparity shift across included classes is as small as possible $\left({\text{Avg}}_{I_{S_{k}}}=-1\right)$. In this case:


$$\begin{array}{c} {\text{Deceptive Signal}}_{S_{k}}=1-(-1)=2. \end{array}$$

Similarly, the minimum value occurs when the disparity shift for the excluded class is at its lowest $\left(\Delta DP_{C_{ex},S_{k}}=-1\right)$ and the average disparity shift across included classes is at its highest $\left({\text{Avg}}_{S_{k}}=1\right)$, giving:


$$\begin{array}{c} {\text{Deceptive Signal}}_{S_{k}}=-1-1=-2. \end{array}$$

Thus, in general, the bias metric is bounded by:


Deceptive SignalSk∈[−2,2]

This range is universal and applies to all datasets and scenarios, regardless of the specific values of *DP*^Partial^ and *DP*^Full^.

To interpret these values in the context of model reliability, a Deceptive Signal near 0 indicates little or no differential shift under the proposed exclusion-inclusion setup, where the introduction of center-specific samples does not cause the model to shift its decision logic toward spurious shortcuts. Values transitioning toward the upper bound of +2 indicate an increasing reliance on deceptive features, where the model prioritizes site-specific signatures over task-relevant morphological patterns. Conversely, while negative values toward −2 are mathematically possible, they represent an inverse effect where partial exposure improves performance on excluded classes more than included ones, a scenario that is rare in practice but defines the lower mathematical limit of the metric.

### 3.3. Probabilistic method justification

This section justifies the Deceptive Bias Detection method using a probabilistic fairness framework. We aim to determine whether a model relies on sensitive subgroup-specific patterns (shortcuts) by comparing its behavior when such patterns are fully excluded versus when they are partially observed during training.

We define two training configurations:

- Full Exclusion: The model is trained without any samples from subgroup $S_{k}$.

$$\begin{array}{c} D_{\text{train}}^{\text{Full}}=D⧵S_{k}\Rightarrow x\in D_{\text{train}}^{\text{Full}}\Rightarrow x\notin (C_{ex}\cap S_{k}) \end{array}$$ (6)

- Partial Exclusion: The model is trained with all samples from $S_{k}$ except those from class $C_{ex}$.

$$\begin{array}{c} D_{\text{train}}^{\text{Partial}}=D⧵(C_{ex}\cap S_{k})\Rightarrow x\in D_{\text{train}}^{\text{Partial}}\Rightarrow x\notin (C_{ex}\cap S_{k}) \end{array}$$ (7)

In both training datasets, $D_{\text{train}}^{\text{Full}}$ and $D_{\text{train}}^{\text{Partial}}$, the model remains unexposed to samples from the intersection $\left(C_{ex},S_{k}\right)$.

We now define two prediction distributions corresponding to the two exclusion settings:

- $P(f(x)|\sim S_{k})$: The prediction behavior when the model is trained with no samples from $S_{k}$.
- $P(f(x)|\sim (S_{k}\wedge C_{ex}))$: The prediction behavior when the model is trained with selective access to $S_{k}$, excluding only samples from $C_{ex}$.

**Fairness condition.** If the model’s prediction behavior is insensitive to subgroup-specific shortcut patterns under this setup, then excluding the entirety of $S_{k}$ or only the subset $\left(C_{ex}\cap S_{k}\right)$ should not alter its behavior on test samples $x\in (C_{ex},S_{k})$. Under this condition, we should observe:


$$\begin{array}{c} P(f(x)|\sim S_{k})=P(f(x)|\sim (S_{k}\wedge C_{ex})) \end{array}$$ (8)

This equality is based on the assumption of conditional independence under fairness, which posits that once we account for the true class $C_{ex}$, the model’s prediction should be independent of the sensitive attribute $S_{k}$. Formally, this is expressed as:


$$\begin{array}{c} \text{If fairness holds}\Rightarrow f(x)⟂S_{k}|C_{ex} \end{array}$$ (9)

This means that, given the same class label, the subgroup from which a sample originates should not affect the model’s prediction. In a fair model, predictions are driven exclusively by class-relevant features. Therefore, even when partial exposure to $S_{k}$ is allowed (excluding only *C*_ex_), the model’s behavior on unseen samples from $\left(C_{ex},S_{k}\right)$ should remain consistent with the full exclusion case. However, if the model has learned shortcut features or latent signatures from remaining classes within $S_{k}$ in the partial exclusion setting, then its behavior will shift, resulting in:


$$\begin{array}{c} P(f(x)|\sim S_{k})\neq P(f(x)|\sim (S_{k}\wedge C_{ex})) \end{array}$$ (10)

### 3.4. Case study

To demonstrate the practical effectiveness of the Deceptive Bias Detection method, we conducted a case study using the Cancer Genome Atlas (TCGA) histopathology dataset. The primary objective was to investigate whether deep learning models rely on non-task-relevant patterns, specifically data center-specific signatures, when performing cancer classification. The TCGA dataset [40] contains whole-slide histopathology images collected from multiple medical centers, each labeled with a cancer type. Prior research has shown that deep models trained on TCGA to learn cancerous features can unintentionally discriminate data-site origin via spurious features [11,12]. To this end, we apply our methodology to this test case to investigate the reliability of the learning process across various deep architectures.

In our experiments, we evaluate three widely used convolutional neural network (CNN) architectures: EfficientNet [41], DenseNet121 [42], and ResNet50V2 [43]. These models are fine-tuned on various training subsets designed according to the Deceptive Bias Detection framework to assess whether they exploit center-based artifacts as shortcuts.

#### 3.4.1. A summary of the dataset and deep models.

The raw TCGA dataset contains 32,072 whole-slide images (WSIs) originating from 156 medical centers across 30 cancer types [44]. However, not all WSIs in the repository are diagnostic slides; many consist of frozen sections or non-representative tissue that require extensive filtering and preprocessing. To ensure a robust and controlled study, we focused exclusively on two cancer types: Lung Adenocarcinoma (LUAD) and Lung Squamous Cell Carcinoma (LUSC). These types were selected because high-quality, preprocessed diagnostic slides were already available, allowing for a more precise analysis of shortcut learning. The samples were selected from a diverse set of medical centers, including Asterand, Prince Charles Hospital, Roswell Park, Johns Hopkins (JHU), and University of Pittsburgh (Pitt), which were utilized in different configurations as discussed in the following subsection.

For our analysis, each WSI was scanned at 20× magnification and partitioned into patches of 1000×1000 pixels. Low-quality or non-diagnostic regions were excluded following established protocols [44,45]. To prepare the input for the models, each patch was further split into overlapping tiles of 224×224 pixels to match the input requirements of our architectures. During the training phase, we employed a transfer learning strategy by initializing the models with ImageNet weights and freezing the early layers to preserve low-level feature representations while allowing domain-specific fine-tuning on the final layers.

**Fig 1:**
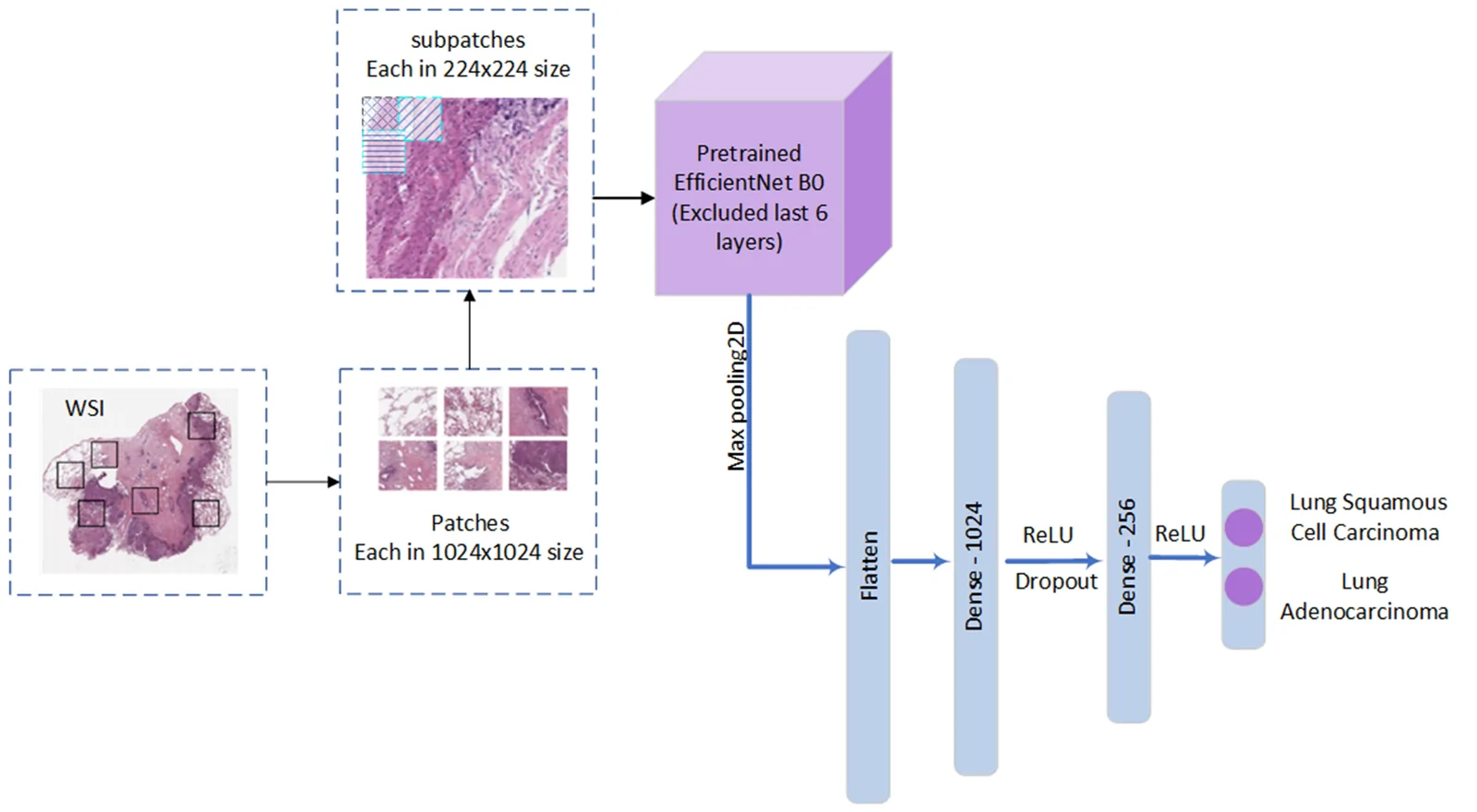
An overview of the input processing and model adaptation strategy. Input patches are partitioned into sub-patches compatible with the EfficientNet architecture. During transfer learning, model adaptation is restricted to the final six layers while the remaining weights are kept frozen to preserve pretrained feature representations.

Specifically, the depth of fine-tuning was adjusted for each architecture: for EfficientNet, all layers were frozen except for the final six; for ResNet50V2, the last 15 layers remained trainable; and for DenseNet121, we permitted training on the final 10 layers. The specific implementation for the EfficientNet-B0 model is depicted in Fig 1 and the detailed code for all three architectures is provided in our GitHub repository to ensure transparency and reproducibility.

#### 3.4.2. Experimental setup.

To ensure the reproducibility and statistical robustness of our findings, we conducted all experiments using five distinct random seeds. In deep learning, the specific choice of a random seed influences several critical aspects of the training process, including the stochastic initialization of model weights, the sequence of data shuffling, and the behavior of dropout layers. By averaging results across multiple seeds, we were able to calculate the mean and standard deviation for all performance metrics, thereby mitigating the impact of stochastic variability.

For each experimental configuration, the dataset was partitioned into training, validation, and testing sets with an initial ratio of 80:10:10. However, to facilitate our deceptive bias analysis, we implemented a specific center-based partitioning strategy. Unlike standard random splits, our test sets consisted exclusively of data from the target center under investigation, containing balanced samples from both cancer classes (LUAD and LUSC). This ensures that the model’s performance is evaluated strictly on its ability to generalize to an unseen center or a partially seen center, depending on the phase of the pipeline.

To evaluate the metric’s performance across varying levels of data diversity, we categorized the experiments into three primary configurations. In each scenario, a target center was designated for deceptive analysis, acting as the unseen or partially excluded subgroup ($S_{k}$).

- **3-Center Configuration:** We utilized data from Asterand, Prince Charles Hospital, and Roswell Park; experiments were conducted twice, designating Asterand and Roswell Park as the target centers in separate runs.
- **4-Center Configuration:** We utilized data from Johns Hopkins University (JHU), Prince Charles Hospital, Roswell Park, and Asterand; analysis was performed using Asterand and Johns Hopkins University as the target centers in separate runs.
- **5-Center Configuration:** We utilized data from Prince Charles Hospital, Roswell Park, Asterand, JHU, and the University of Pittsburgh; the analysis targeted a combined test set composed of samples evenly distributed from both Johns Hopkins University and the University of Pittsburgh.

## 4. Results

In this section, we present the experimental validation of the Deceptive Signal (*S*) metric across diverse experimental settings. To ensure clarity, we first provide an illustrative walkthrough of the experimental procedure using a single 3-center configuration, detailing how behavioral shifts are calculated through the three phases of the pipeline. Subsequently, we present comprehensive results across multiple architectures (EfficientNet, ResNet, and DenseNet) and cross-institutional validation experiments (3, 4, and 5 centers). Finally, we conclude with a statistical significance analysis to assess the robustness of the observed deceptive bias.

### 4.1. Experimental procedure on TCGA

To clarify the implementation details of the Deceptive Pipeline and demonstrate how the final bias signal is derived, we provide a detailed walkthrough using the 3-center configuration. By focusing on a single target center, we can trace the behavioral shifts of the model as it transitions from a state of no exposure to the target center during training to partial exposure to site-specific artifacts.

First, we examine how origin-specific signatures in samples from Asterand may influence the reliability and fairness of deep learning models. For clarity, we present each step of the proposed method in detail.

**Fig 2:**
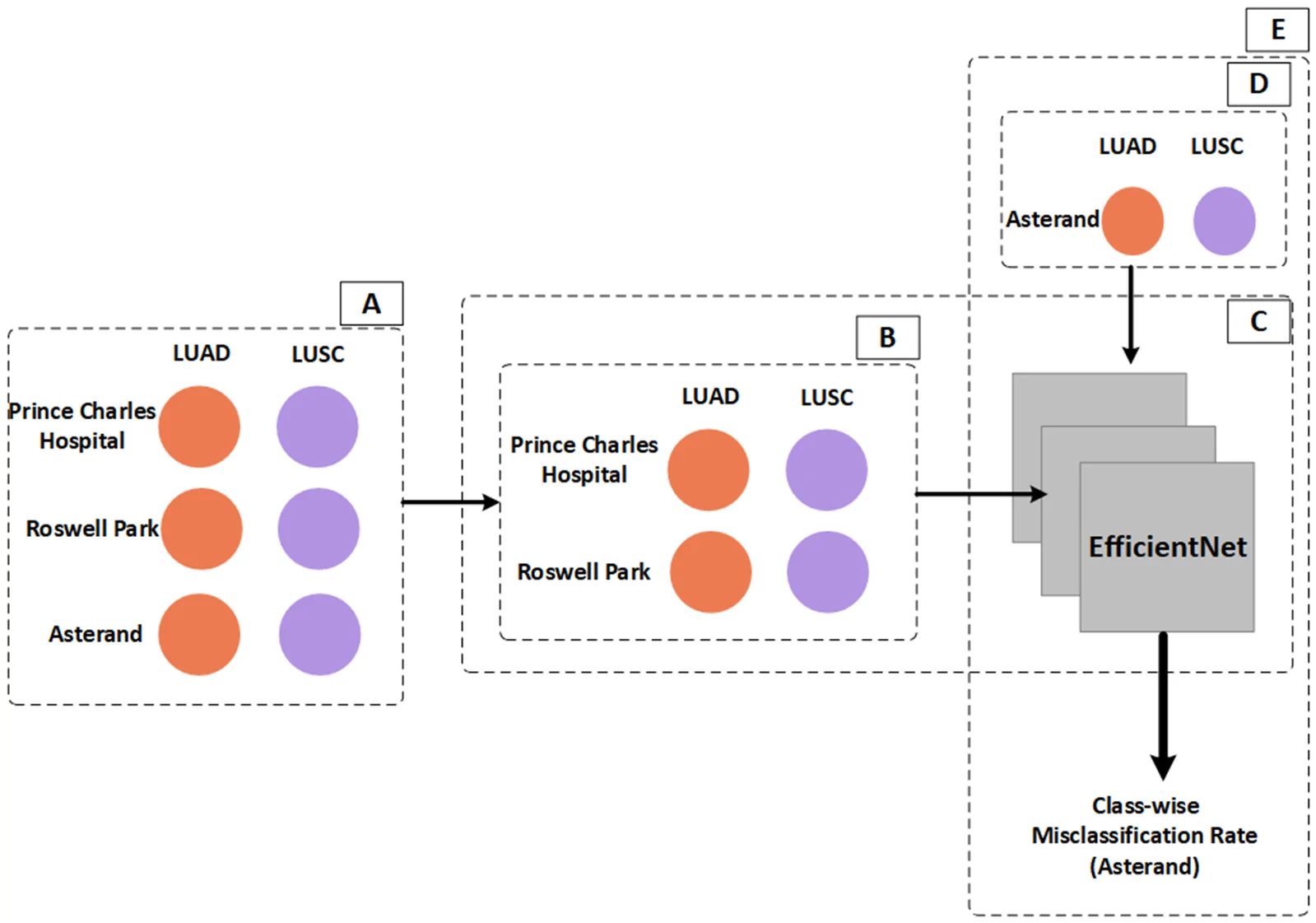
An overview of the full shortcut exclusion scenario. (A) The complete dataset includes LUAD and LUSC samples from three medical centers. (B) The training set is constructed by excluding all Asterand samples. (C) EfficientNet is trained only on Prince Charles and Roswell Park data. (D) The model is then tested on samples from the Asterand data center across both classes. (E) The output reports the misclassification rate for each class, defined as the number of misclassified samples relative to the total samples per class.

**Phase 1. Full Exclusion.** Initially, we exclude all data samples corresponding to $S_{k}=Asterand$ from the training set, defining the training distribution as ${𝒟}_{\text{train}}=𝒟⧵\{Asterand\}$. Then, we use the remaining data to fine-tune the model. Notably, the samples of the other two data centers, which are evenly distributed across both cancer types, remain fixed throughout all training steps to ensure a consistent baseline; the procedure is outlined in Fig 2.


$$\begin{array}{c} f_{-Asterand}\leftarrow \text{FineTune}(f_{\text{pretrained}},{𝒟}_{\text{train}}) \end{array}$$ (11)

Next, we apply the fine-tuned model to the data samples of both cancer classes from the Asterand data center.


$$\begin{array}{c} \hat{y}=f_{-Asterand}(x),\;\forall x\in {𝒟}_{\text{test}}^{\text{LUAD},Asterand}\cup {𝒟}_{\text{test}}^{\text{LUSC},Asterand} \end{array}$$ (12)

Then, for each cancer class (LUAD and LUSC) included in ${𝒟}_{\text{test}}^{Asterand}$, we calculate the proportion of samples that are misclassified as the other class. We consider the results achieved in this step (representing the complete removal of data samples from the Asterand data center) as the baseline to conduct further experiments.

The misclassification rate or disparity proportion for LUAD and LUSC cancer types is calculated as shown in Eqs. 13 and 14:


$$\begin{array}{c} DP_{LUAD,Asterand}^{full}=\left(\frac{48}{\text{256}}\right)=0.19 \end{array}$$ (13)

$$\begin{array}{c} DP_{LUSC,Asterand}^{full}=\left(\frac{149}{\text{257}}\right)=0.58 \end{array}$$ (14)

**Fig 3:**
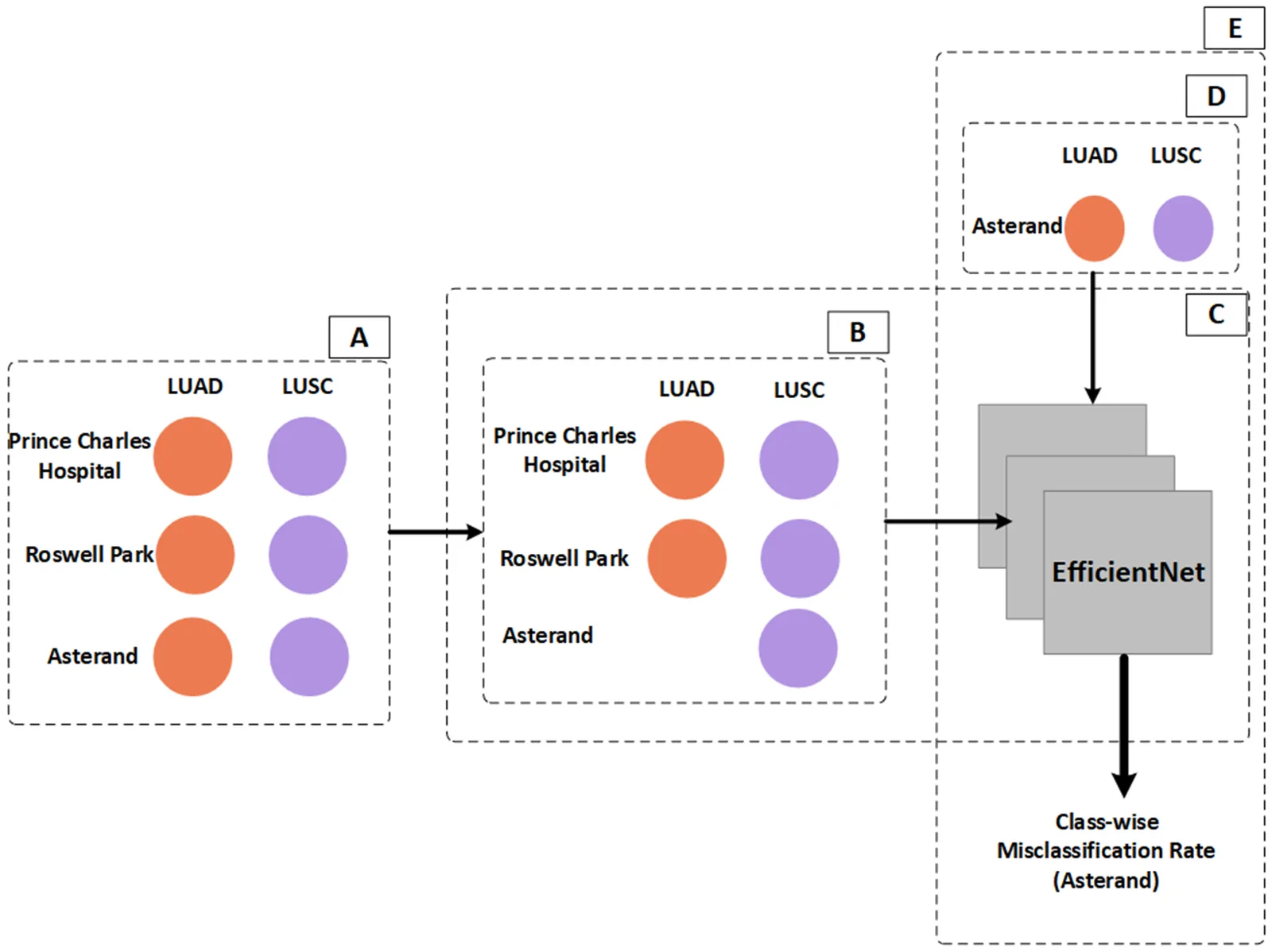
An overview of the partial shortcut exclusion scenario. (A) The complete dataset includes LUAD and LUSC samples from three medical centers. (B) The training set is constructed by excluding only the LUAD class from the Asterand center. (C) EfficientNet is trained only on Prince Charles and Roswell Park data plus the LUSC class from Asterand. (D) The model is then tested on samples from the Asterand data center across both classes. (E) The output reports the misclassification rate for each class, defined as the number of misclassified samples.

**Phase 2. Partial Exclusion**. In this phase, referred to as partial data exclusion (outlined in Fig 3), we do not exclude all samples from the Asterand data center. Instead, we exclude only one cancer class from Asterand, while retaining the remaining classes from the same center in the training set. Specifically, we decide to exclude LUAD samples from Asterand, ${𝒟}_{\text{train}}=𝒟⧵\{Asterand\cap LUAD\}$. Next, we fine-tune the deep model using this modified dataset. In this setup, the model is exposed to only one cancer class from the Asterand data center (LUSC), along with both cancer classes from the other two centers. For evaluation, we use the same test set as in the previous step, which includes samples from both cancer types originating from Asterand, to calculate the disparity proportion. This allows us to observe how this partial exclusion affects the model’s performance over the same test samples of the LUAD class.


$$\begin{array}{c} DP_{LUAD,Asterand}^{Partial}=\left(\frac{198}{\text{256}}\right)=0.77 \end{array}$$ (15)

$$\begin{array}{c} \Delta DP_{LUAD,Asterand}=DP_{LUAD,Asterand}^{\text{Partial}}-DP_{LUAD,Asterand}^{\text{Full}} \end{array}$$ (16)

$$\begin{array}{c} \Delta DP_{LUAD,Asterand}=0.77-0.19=0.58 \end{array}$$ (17)

In this step, we observe that when the LUSC class from Asterand is included and the LUAD samples from this center are excluded, the number of LUAD samples misclassified as LUSC (the included class) increases significantly, from 48 to 198, as presented in Eqs 13 and 15. This indicates that during training, the model captures center-specific signatures embedded in the included class, LUSC. As a result, when evaluating the model on unseen cancer types from the same center, it may rely on these spurious signatures for prediction, leading to incorrect classifications. This phenomenon mirrors the real-world example of a teacher designing exam questions. Modifying the training sample structure is similar to adjusting the tutorial questions used during instruction. The goal is to test whether students truly understand the underlying concepts or have merely memorized specific examples. In the subsequent analysis, we evaluated the misclassified samples for the LUSC class after including its samples in the training data. Only two test samples were misclassified, showing that the inclusion of the LUSC class from the Asterand center significantly reduced misclassification compared to full exclusion.


$$\begin{array}{c} DP_{LUSC,Asterand}^{Partial}=\left(\frac{2}{257}\right)=0.007 \end{array}$$ (18)

The corresponding ΔDP for this class is:


$$\begin{array}{c} \Delta DP_{LUSC,Asterand}=0.007-0.58=-0.573 \end{array}$$ (19)

**Phase 3. Deceptive Signal Measurement.** We compare the disparity proportions under full and partial exclusion to quantify the reliance on center-specific shortcuts. By definition, $\Delta DP=DP^{\text{Partial}}-DP^{\text{Full}}$. For the LUAD class in the Asterand center, the misclassification rate increased sharply from 0.19 under full exclusion (Eq. 13) to 0.77 under partial exclusion (Eq. 15), yielding $\Delta DP_{LUAD,Asterand}=0.58$. This indicates that the model may rely on center-specific signatures captured from the included LUSC class to classify LUAD samples, rather than learning cancer-relevant features.

In contrast, for the LUSC class, the misclassification rate dropped from 0.58 in the full exclusion setting (Eq. 14) to only 0.007 after partial inclusion (Eq. 18), giving $\Delta DP_{LUSC,Asterand}=-0.573$. This shows that including the LUSC class during training reduced errors significantly, as the model had direct access to relevant class samples from Asterand.

Since we have only one included class, the baseline is set as ${\text{Avg}}_{I_{\text{Asterand}}}=\Delta DP_{LUSC,Asterand}=-0.573$. The Deceptive Signal for LUAD is then:


$$\begin{array}{c} {\text{Deceptive Signal}}_{Asterand}=\Delta DP_{LUAD,Asterand}-{\text{Avg}}_{I_{Asterand}}=0.58-(-0.573)=1.153. \end{array}$$

This large positive value (within the theoretical range [−2,2]) highlights a substantial deceptive effect: although including LUSC reduced misclassification for that class, it simultaneously biased predictions for the excluded LUAD class toward Asterand-specific patterns rather than task-relevant cancer features.

### 4.2. Cross-architecture performance and statistical synthesis

In this section, we present a comprehensive evaluation of the Deceptive Signal across all experimental configurations. To improve reliability and assess variability in our findings, each reported value represents the mean and standard deviation calculated across five independent runs.

The following tables summarize the misclassification rates (*DP*) and the resulting Deceptive Signals (*S*) across the 3-center, 4-center, and 5-center configurations. To facilitate a comparative analysis across different architectural backbones, we present the results for each model independently: Table 1 details the performance of EfficientNet-B0, Table 2 summarizes ResNet50V2, and Table 3 provides the results for DenseNet121. These results collectively demonstrate how the inclusion of site-specific signatures consistently biases the models, regardless of their underlying architecture.

**Table 1:** Assessment of Deceptive Shortcuts within the EfficientNet Model. Performance shifts (*DP*) are reported for Full Exclusion (baseline) vs. Rotating Partial Exclusion (*C*_0_ or *C*_1_ exposure). The Deceptive Signal (*S*) quantifies shortcut reliance by measuring behavioral discrepancies on unseen domain classes. Data represent mean ± SD (*n* = 5); strong signals in the JHU cohort (S≈1.0) indicate that EfficientNet is highly susceptible to site-specific background artifacts.

Config	Center	Full Exclusion		Partial (Excl. *C*_0_)			Partial (Excl. *C*_1_)		
		Class	*DP* ^Full^	Class	*DP* ^Partial^	Signal	Class	*DP* ^Partial^	Signal
3 Centers	Asterand	*C*_0_	0.63±0.05	*C*_0_	0.70±0.05	0.13±0.08	*C*_0_	0.10±0.05	1.01±0.11
		*C*_1_	0.1±0.03	*C*_1_	0.03±0.02		*C*_1_	0.57±0.08	
	Roswell Park	*C*_0_	0.37±0.11	*C*_0_	0.60±0.01	0.45±0.18	*C*_0_	0.05±0.02	0.48±0.15
		*C*_1_	0.30±0.08	*C*_1_	0.09±0.05		*C*_1_	0.46±0.06	
4 Centers	Asterand	*C*_0_	0.46±0.08	*C*_0_	0.69±0.09	0.42±0.14	*C*_0_	0.17±0.05	0.50±0.13
		*C*_1_	0.23±0.07	*C*_1_	0.05±0.02		*C*_1_	0.44±0.06	
	JHU	*C*_0_	0.50±0.05	*C*_0_	0.75±0.09	0.35±0.11	*C*_0_	0.07±0.04	1.00±0.10
		*C*_1_	0.14±0.03	*C*_1_	0.04±0.02		*C*_1_	0.71±0.07	
5 Centers	Pitt & JHU	*C*_0_	0.52±0.07	*C*_0_	0.71±0.02	0.30±0.09	*C*_0_	0.16±0.07	0.67±0.14
		*C*_1_	0.20±0.04	*C*_1_	0.10±0.03		*C*_1_	0.52±0.10	

**Table 2:** Assessment of Deceptive Shortcuts within the ResNet Model. Performance shifts (*DP*) are reported for Full Exclusion (baseline) vs. Rotating Partial Exclusion (*C*_0_ or *C*_1_ exposure). The Deceptive Signal (*S*) quantifies shortcut reliance by measuring behavioral discrepancies on unseen domain classes. Data represent mean ± SD (*n* = 5); the peak signal of 1.15 in the Asterand setting reveals that ResNet adopts shortcuts even when provided with more diverse training data.

Config	Center	Full Exclusion		Partial (Excl. *C*_0_)			Partial (Excl. *C*_1_)		
		Class	*DP* ^Full^	Class	*DP* ^Partial^	Signal	Class	*DP* ^Partial^	Signal
3 Centers	Asterand	*C*_0_	0.50±0.12	*C*_0_	0.71±0.07	1.15±0.05	*C*_0_	0.12±0.07	0.73±0.10
		*C*_1_	0.26±0.13	*C*_1_	0.10±0.03		*C*_1_	0.61±0.15	
	Roswell Park	*C*_0_	0.37±0.09	*C*_0_	0.73±0.08	0.71±0.15	*C*_0_	0.06±0.03	0.47±0.14
		*C*_1_	0.42±0.09	*C*_1_	0.07±0.02		*C*_1_	0.58±0.05	
4 Centers	Asterand	*C*_0_	0.51±0.19	*C*_0_	0.58±0.12	0.18±0.03	*C*_0_	0.17±0.14	0.6±0.36
		*C*_1_	0.27±0.18	*C*_1_	0.16±0.08		*C*_1_	0.54±0.2	
	JHU	*C*_0_	0.48±0.16	*C*_0_	0.66±0.02	0.3±0.02	*C*_0_	0.15±0.05	0.65±0.02
		*C*_1_	0.19±0.11	*C*_1_	0.07±0.03		*C*_1_	0.51±0.08	
5 Centers	Pitt & JHU	*C*_0_	0.53±0.12	*C*_0_	0.7±0.09	0.35±0.17	*C*_0_	0.15±0.08	0.71±0.19
		*C*_1_	0.30±0.08	*C*_1_	0.12±0.03		*C*_1_	0.62±0.1	

**Table 3:** Assessment of Deceptive Shortcuts within the DenseNet Model. Performance shifts (*DP*) are reported for Full Exclusion (baseline) vs. Rotating Partial Exclusion (*C*_0_ or *C*_1_ exposure). The Deceptive Signal (*S*) quantifies shortcut reliance by measuring behavioral discrepancies on unseen domain classes. Data represent mean ± SD (*n* = 5); DenseNet shows the most balanced susceptibility across centers, yet maintains statistically significant deceptive signals (*p* < 0.05) in all 3, 4, and 5-center settings.

Config	Center	Full Exclusion		Partial (Excl. *C*_0_)			Partial (Excl. *C*_1_)		
		Class	*DP* ^Full^	Class	*DP* ^Partial^	Signal	Class	*DP* ^Partial^	Signal
3 Centers	Asterand	*C*_0_	0.54±0.09	*C*_0_	0.74±0.16	0.33±0.21	*C*_0_	0.09±0.07	0.83±0.20
		*C*_1_	0.20±0.07	*C*_1_	0.07±0.08		*C*_1_	0.58±0.15	
	Roswell Park	*C*_0_	0.37±0.10	*C*_0_	0.71±0.09	0.62±0.17	*C*_0_	0.04±0.03	0.50±0.17
		*C*_1_	0.32±0.10	*C*_1_	0.04±0.02		*C*_1_	0.49±0.08	
4 Centers	Asterand	*C*_0_	0.55±0.13	*C*_0_	0.70±0.13	0.25±0.22	*C*_0_	0.11±0.06	0.84±0.23
		*C*_1_	0.16±0.11	*C*_1_	0.06±0.05		*C*_1_	0.55±0.15	
	JHU	*C*_0_	0.45±0.10	*C*_0_	0.63±0.08	0.33±0.16	*C*_0_	0.07±0.04	0.88±0.16
		*C*_1_	0.22±0.09	*C*_1_	0.07±0.04		*C*_1_	0.71±0.08	
5 Centers	Pitt & JHU	*C*_0_	0.54±0.08	*C*_0_	0.78±0.10	0.41±0.15	*C*_0_	0.11±0.05	0.76±0.26
		*C*_1_	0.22±0.06	*C*_1_	0.05±0.04		*C*_1_	0.55±0.24	

The results presented in Tables 1–3 suggest reduced generalization when models are exposed to partial site-specific data. Across all three architectures, we observe a consistent deceptive shift; while the inclusion of a single class from a target center ($S_{k}$) drastically reduces misclassification for that specific class ($DP^{\text{Partial}}\approx 0$), it simultaneously inflates the error rate for the excluded class within the same center. This divergence indicates that the models are not learning invariant biological features of LUAD or LUSC; instead, they are performing a shortcut lookup by identifying center-specific artifacts and mapping them to the only available label seen during training. The high magnitude of the Deceptive Signal (*S*), often exceeding 1.0, quantifies this reliance, suggesting that the model’s perceived accuracy at a specific site is largely an artifact of encoded site bias rather than task-relevant diagnostic features.

In Table 4, we provide a comprehensive synthesis of the deceptive signals across all three architectures and experimental configurations to evaluate the consistency of shortcut learning. By combining the results from all center configurations, we show that this deceptive effect is not a one-time error; rather, it is a consistent weakness found across all three models: EfficientNet, ResNet, and DenseNet. The table highlights that while the magnitude of the signal fluctuates across different target centers and models, a significant positive signal persists in nearly every configuration. This cross-architecture comparison ultimately supports that the reliance on center-specific artifacts is a fundamental characteristic of these deep learning models rather than an artifact of a specific network design.

**Table 4:** A cross-architecture synthesis of the Deceptive Signal (*S*) across multi-center configurations. Bolded values highlight instances of high shortcut reliance, where the metric consistently detects bias regardless of model structure or data center rotation. Statistical significance was verified via a one-sample t-test against the null hypothesis *H*_0_: *S* = 0, with most reported signals achieving *p* < 0.05.

Config	Center	Included	EffNet*	ResNet*	DenseNet*	Avg.*
3 Centers	Asterand	*C*_1_ (LUSC)	0.13 ± 0.08	1.15 ± 0.05	0.33 ± 0.21	0.54 ± 0.11
		*C*_0_ (LUAD)	**1.01 ± 0.11**	0.73 ± 0.10	**0.83 ± 0.20**	**0.86 ± 0.14**
	Roswell Park	*C*_1_ (LUSC)	0.45 ± 0.18	**0.71 ± 0.15**	**0.62 ± 0.17**	**0.59 ± 0.17**
		*C*_0_ (LUAD)	0.48 ± 0.15	0.47 ± 0.14	0.50 ± 0.17	0.48 ± 0.15
4 Centers	Asterand	*C*_1_ (LUSC)	0.42 ± 0.14	0.18 ± 0.03	0.25 ± 0.22	0.28 ± 0.13
		*C*_0_ (LUAD)	0.50 ± 0.13	0.60 ± 0.36	0.84 ± 0.23	**0.65 ± 0.24**
	JHU	*C*_1_ (LUSC)	0.35 ± 0.11	0.30 ± 0.02	0.33 ± 0.16	0.33 ± 0.10
		*C*_0_ (LUAD)	**1.00 ± 0.10**	**0.65 ± 0.02**	**0.88 ± 0.16**	**0.84 ± 0.09**
5 Centers	Pitt & JHU	*C*_1_ (LUSC)	0.30 ± 0.09	0.35 ± 0.17	0.41 ± 0.15	0.35 ± 0.14
		*C*_0_ (LUAD)	**0.67 ± 0.14**	**0.71 ± 0.19**	**0.76 ± 0.26**	**0.71 ± 0.20**

* Values represent mean ± standard deviation. Most reported signals are significant at *p* < 0.05 (one-sample t-test against *H*_0_: *S*=0).

### 4.3. Statistical significance analysis

To assess the reliability of the observed deceptive signals, we conducted a statistical assessment of our results. Each experiment was executed across five independent random seeds to assess the robustness of the findings. While variance fluctuated depending on the model architecture and configuration, the calculated standard deviations remained within an acceptable range for deep learning experiments, typically between 0.05 and 0.20. To address the requirement for significance testing, we performed a one-sample t-test against a null hypothesis of *S* = 0 (indicating no deceptive bias). In nearly all configurations, including the primary 3-center Asterand and 4-center JHU scenarios, the results yielded p-values significantly lower than 0.05, indicating that the detected signals are statistically significant and unlikely to be explained solely by stochastic variability. Furthermore, we calculated 95% confidence intervals for these signals; because these intervals consistently do not overlap with the zero-baseline, these results provide evidence that the models may be relying on center-specific shortcuts rather than task-relevant features.

## 5. Discussion

This study introduces the Deceptive Signal metric as a practical method for assessing whether deep learning models are merely learning the task or just memorizing hidden shortcuts. Unlike standard methods that only look at final accuracy scores, our approach tests how the model changes its behavior when we manipulate its training data. The core of this method lies in comparing two specific scenarios: Full Exclusion, where the model sees no data from a sensitive group (like a specific hospital), and Partial Exclusion, where it sees data from only some classes of that group. By observing the difference between these two stages, we can better assess whether a model generalizes or relies on shortcuts.

The logic behind this comparison is straightforward but powerful. In a standard learning process, providing a model with additional data, even from a single class within a specific group, should help it understand the overall domain better. However, our method operates on the opposite hypothesis: if adding partial data actually makes the model perform worse on unseen samples from that same group, it may indicate shortcut-related bias. This suggests that the model did not learn the actual features of the task (like cancer tissue) but instead learned a shortcut, such as the background noise unique to that hospital. This mirrors the math teacher analogy described earlier: if a student memorizes the teacher’s specific exercises during tutorial sessions instead of learning the underlying mathematical principles, they may perform well on similar test questions without truly understanding the subject. Likewise, the Deceptive Signal quantifies the extent to which a model relies on task-irrelevant shortcuts rather than learning the actual objective.

To make this measurement quantitative, we developed a quantitative metric that isolates this specific bias. By subtracting the average behavior of the observed classes from the behavior of the unobserved classes, we ensure that we are measuring the shortcut reliance accurately. This calculation results in a score that is mathematically defined to fall between -2 and 2. This standardized range is a significant advantage because it creates a standardized metric that can be used across different datasets.

Our experiments on the Johns Hopkins data center with a four-center configuration demonstrate this phenomenon. For instance, EfficientNet achieved a Deceptive Signal of 1.00±0.10 for the *C*_0_ (LUAD) class. This suggests that, although the model is intended to learn morphological features to distinguish between cancer types, it is instead substantially influenced by center-specific features, likely arising from variations in staining protocols, slide preparation, or scanning equipment across centers. Such reliance is particularly concerning, as it indicates that the model may fail to generalize to unseen centers where these spurious correlations are absent. The Deceptive Signal is also relatively high for the other two models, with values of 0.65±0.02 and 0.88±0.16, respectively, suggesting that this issue is not architecture-specific but rather inherent to the dataset. These findings highlight the importance of explicitly detecting and mitigating center-induced biases, as conventional performance metrics alone may overestimate a model’s true ability to learn clinically relevant features.

## 6. Conclusion

Despite the utility of the Deceptive Signal (*S*), several constraints must be acknowledged. First, the proposed method relies heavily on the availability of explicit labels for sensitive subgroups, such as the specific data center or hospital where an image was acquired. In many real-world datasets, these metadata may be anonymized, missing, or unreliable, preventing the construction of the necessary exclusion phases. Without these identifiable attributes, isolating specific site-specific shortcuts remains a challenge.

Second, the methodology introduces a higher computational cost compared to traditional fairness metrics. Standard methods typically evaluate bias in a single pass after the model is fully trained. In contrast, our pipeline necessitates multiple training cycles (Full and Partial Exclusion) to quantify the behavioral shift. This requirement for multiple training runs increases the time and computing resources needed, which may be a constraint for large-scale models or resource-limited settings. Finally, while we have evaluated this metric within the high-stakes domain of histopathology, further research is required to test its generalizability across other modalities, such as natural images or structured tabular data.

Looking ahead, we see several promising directions to expand the utility of the Deceptive Signal metric. Although this study focused on medical imaging, a critical next step is to adapt this framework to large language models (LLMs). Like image classifiers, LLMs are prone to learning hidden shortcuts, often associating specific demographic cues or writing styles with certain answers rather than relying on factual content. By applying our exclusion–inclusion strategy to text data, our objective is to verify whether our metric can effectively quantify these reasoning shortcuts in natural language processing, thereby testing the broader applicability of our approach.

Furthermore, we intend to move beyond simple detection and explore mitigation. Currently, our metric serves as a diagnostic tool to identify bias after training. In future research, we plan to integrate the Deceptive Signal calculation directly into the training process itself. By using this metric as a penalty term in the loss function, we could force the model to unlearn spurious correlations in real time, effectively creating a shortcut-averse learning algorithm.

## Supporting information

S1 Data Raw experimental results for the Deceptive Signal analysis. The file contains seed-level baseline and partial-exclusion error rates for LUAD and LUSC across EfficientNet-B0, ResNet50V2, and DenseNet121 under the 3-center, 4-center, and 5-center experimental configurations.
(PDF)

## References

[1] Shah M, Sureja N. A Comprehensive Review of Bias in Deep Learning Models: Methods, Impacts, and Future Directions. Arch Computat Methods Eng. 2024;32(1):255–67. doi: 10.1007/s11831-024-10134-2

[2] Nazer LH, Zatarah R, Waldrip S, Ke JXC, Moukheiber M, Khanna AK, et al. Bias in artificial intelligence algorithms and recommendations for mitigation. PLOS Digit Health. 2023;2(6):e0000278. doi: 10.1371/journal.pdig.0000278 37347721 PMC10287014

[3] Rabonato RT, Berton L. A systematic review of fairness in machine learning. AI Ethics. 2024;5(3):1943–54. doi: 10.1007/s43681-024-00577-5

[4] Mehrabi N, Morstatter F, Saxena N, Lerman K, Galstyan A. A Survey on Bias and Fairness in Machine Learning. ACM Comput Surv. 2021;54(6):1–35. doi: 10.1145/3457607

[5] Geirhos R, Jacobsen J-H, Michaelis C, Zemel R, Brendel W, Bethge M, et al. Shortcut learning in deep neural networks. Nat Mach Intell. 2020;2(11):665–73. doi: 10.1038/s42256-020-00257-z

[6] Nauta M, Walsh R, Dubowski A, Seifert C. Uncovering and Correcting Shortcut Learning in Machine Learning Models for Skin Cancer Diagnosis. Diagnostics (Basel). 2021;12(1):40. doi: 10.3390/diagnostics12010040 35054207 PMC8774502

[7] Xu Z, Xiang X, Liang Y. Overcoming Shortcut Problem in VLM for Robust Out-of-Distribution Detection. In: 2025 IEEE/CVF Conference on Computer Vision and Pattern Recognition (CVPR), 2025. 15402–12. 10.1109/cvpr52734.2025.01435

[8] Kheiri F, Rahnamayan S, Makrehchi M, Asilian Bidgoli A. Investigation on potential bias factors in histopathology datasets. Sci Rep. 2025;15(1):11349. doi: 10.1038/s41598-025-89210-x 40175463 PMC11965531

[9] Zhao L, Liu Q, Yue L, Chen W, Chen L, Sun R, et al. COMI: COrrect and MItigate Shortcut Learning Behavior in Deep Neural Networks. In: Proceedings of the 47th International ACM SIGIR Conference on Research and Development in Information Retrieval, 2024. 218–28. 10.1145/3626772.3657729

[10] Cross JL, Choma MA, Onofrey JA. Bias in medical AI: Implications for clinical decision-making. PLOS Digit Health. 2024;3(11):e0000651. doi: 10.1371/journal.pdig.0000651 39509461 PMC11542778

[11] Kheiri F, Bidgoli AA, Makrehchi M, Rahnamayan S. Feature Selection-driven Bias Deduction in Histopathology Images: Tackling Site-Specific Influences. In: 2024 IEEE Congress on Evolutionary Computation (CEC), 2024. 1–8. 10.1109/cec60901.2024.10611837

[12] Dehkharghanian T, Bidgoli AA, Riasatian A, Mazaheri P, Campbell CJV, Pantanowitz L, et al. Biased data, biased AI: deep networks predict the acquisition site of TCGA images. Diagn Pathol. 2023;18(1):67. doi: 10.1186/s13000-023-01355-3 37198691 PMC10189924

[13] Hoche M, Mineeva O, Rätsch G, Vayena E, Blasimme A. What makes clinical machine learning fair? A practical ethics framework. PLOS Digit Health. 2025;4(3):e0000728. doi: 10.1371/journal.pdig.0000728 40100898 PMC11918422

[14] Verma S, Rubin J. Fairness definitions explained. In: Proceedings of the International Workshop on Software Fairness, 2018. 1–7. 10.1145/3194770.3194776

[15] Hermann KL, Mobahi H, Fel T, Mozer MC. On the foundations of shortcut learning. 2023. https://arxiv.org/abs/2310.16228

[16] Selvaraju RR, Das A, Vedantam R, Cogswell M, Parikh D, Batra D. Grad-CAM: Why did you say that?. arXiv preprint. 2016. doi: arXiv:1611.07450

[17] Yang W, Kirichenko P, Goldblum M, Wilson A. Chroma-VAE: Mitigating Shortcut Learning with Generative Classifiers. In: Advances in Neural Information Processing Systems 35, 2022. 20351–65. 10.52202/068431-1480

[18] Guluwadi S. Enhancing brain tumor detection in MRI images through explainable AI using Grad-CAM with Resnet 50. BMC Medical Imaging. 2024;24(1):1–19. 38734629 10.1186/s12880-024-01292-7 PMC11088067

[19] Adebayo J, Gilmer J, Muelly M, Goodfellow I, Hardt M, Kim B. Sanity checks for saliency maps. Advances in Neural Information Processing Systems. 2018;31.

[20] Ullah I, Jian M, Hussain S, Guo J, Yu H, Wang X, et al. A brief survey of visual saliency detection. Multimed Tools Appl. 2020;79(45–46):34605–45. doi: 10.1007/s11042-020-08849-y

[21] Cong R, Lei J, Fu H, Cheng M-M, Lin W, Huang Q. Review of Visual Saliency Detection With Comprehensive Information. IEEE Trans Circuits Syst Video Technol. 2019;29(10):2941–59. doi: 10.1109/tcsvt.2018.2870832

[22] DeGrave AJ, Janizek JD, Lee S-I. AI for radiographic COVID-19 detection selects shortcuts over signal. Nat Mach Intell. 2021;3(7):610–9. doi: 10.1038/s42256-021-00338-7

[23] Dhont J, Wolfs C, Verhaegen F. Automatic coronavirus disease 2019 diagnosis based on chest radiography and deep learning - Success story or dataset bias?. Med Phys. 2022;49(2):978–87. doi: 10.1002/mp.15419 34951033 PMC9015341

[24] Tian B, Guo Q, Juefei-Xu F, Chan WL, Cheng Y, Li X. Bias field poses a threat to dnn-based x-ray recognition. 2020. https://arxiv.org/abs/2009.09247

[25] Adeli E, Zhao Q, Pfefferbaum A, Sullivan EV, Fei-Fei L, Niebles JC, et al. Representation Learning with Statistical Independence to Mitigate Bias. IEEE Winter Conf Appl Comput Vis. 2021;2021:2512–22. doi: 10.1109/wacv48630.2021.00256 34522832 PMC8436589

[26] Boland C, Goatman KA, Tsaftaris SA, Dahdouh S. There are no shortcuts to anywhere worth going: identifying shortcuts in deep learning models for medical image analysis. In: Medical Imaging with Deep Learning, 2024.

[27] Wang R, Kuo P-C, Chen L-C, Seastedt KP, Gichoya JW, Celi LA. Drop the shortcuts: image augmentation improves fairness and decreases AI detection of race and other demographics from medical images. EBioMedicine. 2024;102:105047. doi: 10.1016/j.ebiom.2024.105047 38471396 PMC10945176

[28] Lou J, Wang H, Wu X, Ng JCH, White R, Thakoor KA, et al. Chest X-Ray Visual Saliency Modeling: Eye-Tracking Dataset and Saliency Prediction Model. IEEE Trans Neural Netw Learn Syst. 2025;36(9):16920–30. doi: 10.1109/TNNLS.2025.3564292 40338721

[29] Kusner MJ, Loftus J, Russell C, Silva R. Counterfactual fairness. In: Advances in Neural Information Processing Systems, 2017.

[30] Zhang Y, Sang J, Wang J, Jiang D, Wang Y. Benign Shortcut for Debiasing: Fair Visual Recognition via Intervention with Shortcut Features. In: Proceedings of the 31st ACM International Conference on Multimedia, 2023. 8860–8. 10.1145/3581783.3612317

[31] Dash S, Balasubramanian VN, Sharma A. Evaluating and Mitigating Bias in Image Classifiers: A Causal Perspective Using Counterfactuals. In: 2022 IEEE/CVF Winter Conference on Applications of Computer Vision (WACV), 2022. 3879–88. 10.1109/wacv51458.2022.00393

[32] Vigneshwaran V, Stanley EA, Souza R, Ohara E, Wilms M, Forkert N. Evaluating Shortcut Utilization in Deep Learning Disease Classification through Counterfactual Analysis. In: Medical Imaging with Deep Learning; 2025.

[33] Müller NM, Roschmann S, Khan S, Sperl P, Böttinger K. Shortcut Detection With Variational Autoencoders. In: 2024 International Joint Conference on Neural Networks (IJCNN), 2024. 1–7. 10.1109/ijcnn60899.2024.10650671

[34] Leeb F, Bauer S, Besserve M, Schölkopf B. Exploring the Latent Space of Autoencoders with Interventional Assays. In: Advances in Neural Information Processing Systems 35, 2022. 21562–74. 10.52202/068431-1567

[35] Brown A, Tomasev N, Freyberg J, Liu Y, Karthikesalingam A, Schrouff J. Detecting shortcut learning for fair medical AI using shortcut testing. Nat Commun. 2023;14(1):4314. doi: 10.1038/s41467-023-39902-7 37463884 PMC10354021

[36] Hardt M, Price E, Srebro N. Equality of opportunity in supervised learning. Advances in Neural Information Processing Systems. 2016;29.

[37] Yang Y, Zhang H, Katabi D, Ghassemi M. On mitigating shortcut learning for fair chest x-ray classification under distribution shift. In: 2024.

[38] Yu Z, Chakraborty J, Menzies T. FairBalance: How to Achieve Equalized Odds With Data Pre-Processing. IIEEE Trans Software Eng. 2024;50(9):2294–312. doi: 10.1109/tse.2024.3431445

[39] Jiménez-Sánchez A, Juodelyte D, Chamberlain B, Cheplygina V. Detecting Shortcuts in Medical Images - A Case Study in Chest X-Rays. In: 2023 IEEE 20th International Symposium on Biomedical Imaging (ISBI), 2023. 1–5. 10.1109/isbi53787.2023.10230572

[40] Gutman DA, Cobb J, Somanna D, Park Y, Wang F, Kurc T, et al. Cancer Digital Slide Archive: an informatics resource to support integrated in silico analysis of TCGA pathology data. J Am Med Inform Assoc. 2013;20(6):1091–8. doi: 10.1136/amiajnl-2012-001469 23893318 PMC3822112

[41] Tan M, Le Q. Efficientnet: Rethinking model scaling for convolutional neural networks. In: International conference on machine learning, 2019. 6105–14.

[42] Huang G, Liu Z, Van Der Maaten L, Weinberger KQ. Densely Connected Convolutional Networks. In: 2017 IEEE Conference on Computer Vision and Pattern Recognition (CVPR), 2017. 2261–9. 10.1109/cvpr.2017.243

[43] He K, Zhang X, Ren S, Sun J. Identity Mappings in Deep Residual Networks. Lecture Notes in Computer Science. Springer International Publishing. 2016. p. 630–45. doi: 10.1007/978-3-319-46493-0_38

[44] Riasatian A, Babaie M, Maleki D, Kalra S, Valipour M, Hemati S, et al. Fine-Tuning and training of densenet for histopathology image representation using TCGA diagnostic slides. Med Image Anal. 2021;70:102032. doi: 10.1016/j.media.2021.102032 33773296

[45] Kalra S, Tizhoosh HR, Choi C, Shah S, Diamandis P, Campbell CJV, et al. Yottixel - An Image Search Engine for Large Archives of Histopathology Whole Slide Images. Med Image Anal. 2020;65:101757. doi: 10.1016/j.media.2020.101757 32623275

