# Supplementary material for: Deceptive bias measurement in deep learning: Assessing shortcut reliance in TCGA cancer models
Source: PLOS Digit Health. 2026 Sep 3;5(9):e0001165. doi: 10.1371/journal.pdig.0001165 (PMC13541255; doi:10.1371/journal.pdig.0001165)
Supplement: Supplementary file 1 [file pdig.0001165.s001.pdf]

Table Contents

EfficientNet-B0 Raw Data: 3-Center Setup (Asterand Site) 2

EfficientNet-B0 Raw Data: 3-Center Setup (Roswell Park Site) 3

EfficientNet-B0 Raw Data: 4-Center Setup (Asterand Site) 4

EfficientNet-B0 Raw Data: 4-Center Setup (Johns Hopkins Site)..5

EfficientNet-B0 Raw Data: 5-Center Setup (Johns Hopkins & University of Pittsburgh Sites) 6

RestNet50V2 Raw Data: 3-Center Setup (Asterand Site) 7

RestNet50V2 Raw Data: 3-Center Setup (Roswell Park Site)...8

RestNet50V2 Raw Data: 4-Center Setup (Asterand Site) 9

RestNet50V2 Raw Data: 4-Center Setup (Johns Hopkins Site) 10

RestNet50V2 Raw Data: 5-Center Setup (Johns Hopkins & University of Pittsburgh)......................11

DenseNet121 Raw Data: 3-Center Setup (Asterand Site) 12

DenseNet121 Raw Data: 3-Center Setup (Roswell Park Site)...13

DenseNet121 Raw Data: 4-Center Setup (Asterand Site) 14

DenseNet121 Raw Data: 4-Center Setup (Johns Hopkins Site) 15

DenseNet121 Raw Data: 5-Center Setup (Johns Hopkins & University of Pittsburgh Sites)............16

EfficientNet-B0 Raw Data: 3-Center Setup (Asterand Site)

Table 1. Shortcut Isolation for LUAD

seeds	Baseline - error rare		After Exclusion of LUAD - error rate	
	LUAD	LUSC	LUAD	LUSC
42	0.59	0.1	0.7	0.03
123	0.7	0.07	0.6	0.04
7	0.59	0.15	0.73	0.01
1024	0.57	0.12	0.73	0.07
99	0.68	0.06	0.72	0.02

Table 2. Shortcut Isolation for LUSC

seeds	Baseline - error rare		After Exclusion of LUSC - error rate	
	LUAD	LUSC	LUAD	LUSC
42	0.59	0.1	0.1	0.67
123	0.7	0.07	0.18	0.43
7	0.59	0.15	0.09	0.61
1024	0.57	0.12	0.06	0.58
99	0.68	0.06	0.05	0.58

EfficientNet-B0 Raw Data: 3-Center Setup (Roswell Park Site)

Table 3. Shortcut Isolation for LUAD

seeds	Baseline - error rare		After Exclusion of LUAD - error rate	
	LUAD	LUSC	LUAD	LUSC
42	0.24	0.39	0.72	0.04
123	0.55	0.19	0.59	0.12
7	0.32	0.34	0.55	0.17
1024	0.32	0.35	0.73	0.08
99	0.43	0.23	0.43	0.06

Table 4. Shortcut Isolation for LUSC

seeds	Baseline - error rare		After Exclusion of LUSC - error rate	
	LUAD	LUSC	LUAD	LUSC
42	0.24	0.39	0.003	0.57
123	0.55	0.19	0.07	0.19
7	0.32	0.34	0.06	0.4
1024	0.32	0.35	0.07	0.58
99	0.43	0.23	0.05	0.45

EfficientNet-B0 Raw Data: 4-Center Setup (Asterand Site)

Table 5. Shortcut Isolation for LUAD

seeds	Baseline - error rare		After Exclusion of LUAD - error rate	
	LUAD	LUSC	LUAD	LUSC
42	0.33	0.34	0.77	0.05
123	0.57	0.14	0.63	0.05
7	0.42	0.22	0.8	0.03
1024	0.49	0.19	0.56	0.08
99	0.47	0.24	0.71	0.03

Table 6. Shortcut Isolation for LUSC

seeds	Baseline - error rare		After Exclusion of LUSC - error rate	
	LUAD	LUSC	LUAD	LUSC
42	0.33	0.34	0.14	0.49
123	0.57	0.14	0.25	0.33
7	0.42	0.22	0.11	0.49
1024	0.49	0.19	0.16	0.45
99	0.47	0.24	0.17	0.43

EfficientNet-B0 Raw Data: 4-Center Setup (Johns Hopkins Site)

Table 7. Shortcut Isolation for LUAD

seeds	Baseline - error rare		After Exclusion of LUAD - error rate	
	LUAD	LUSC	LUAD	LUSC
42	0.53	0.1	0.63	0.07
123	0.47	0.14	0.69	0.03
7	0.5	0.16	0.72	0.03
1024	0.43	0.18	0.86	0.02
99	0.58	0.12	0.7	0.05

Table 8. Shortcut Isolation for LUSC

seeds	Baseline - error rare		After Exclusion of LUSC - error rate	
	LUAD	LUSC	LUAD	LUSC
42	0.53	0.1	0.05	0.76
123	0.47	0.14	0.03	0.76
7	0.5	0.16	0.07	0.69
1024	0.43	0.18	0.13	0.58
99	0.58	0.12	0.06	0.75

EfficientNet-B0 Raw Data: 5-Center Setup (Johns Hopkins & University of Pittsburgh Sites)

Table 9. Shortcut Isolation for LUAD

seeds	Baseline - error rare		After Exclusion of LUAD - error rate	
	LUAD	LUSC	LUAD	LUSC
42	0.44	0.27	0.73	0.08
123	0.53	0.22	0.69	0.07
7	0.48	0.17	0.7	0.13
1024	0.52	0.18	0.70	0.13
99	0.64	0.14	0.74	0.07

Table 10. Shortcut Isolation for LUSC

seeds	Baseline - error rare		After Exclusion of LUSC - error rate	
	LUAD	LUSC	LUAD	LUSC
42	0.44	0.27	0.17	0.47
123	0.53	0.22	0.19	0.49
7	0.48	0.17	0.06	0.66
1024	0.52	0.18	0.26	0.37
99	0.64	0.14	0.13	0.59

RestNet50V2 Raw Data: 3-Center Setup (Asterand Site)

Table 11. Shortcut Isolation for LUAD

seeds	Baseline - error rare		After Exclusion of LUAD - error rate	
	LUAD	LUSC	LUAD	LUSC
42	0.5	0.25	0.76	0.08
123	0.65	0.14	0.65	0.11
7	0.29	0.51	0.82	0.06
1024	0.47	0.21	0.68	0.15
99	0.57	0.2	0.66	0.09

Table 12. Shortcut Isolation for LUSC

seeds	Baseline - error rare		After Exclusion of LUSC - error rate	
	LUAD	LUSC	LUAD	LUSC
42	0.5	0.25	0.08	0.72
123	0.65	0.14	0.09	0.64
7	0.29	0.51	0.16	0.46
1024	0.47	0.21	0.24	0.41
99	0.57	0.2	0.04	0.8

RestNet50V2 Raw Data: 3-Center Setup (Roswell Park Site)

Table 13. Shortcut Isolation for LUAD

seeds	Baseline - error rare		After Exclusion of LUAD - error rate	
	LUAD	LUSC	LUAD	LUSC
42	0.4	0.4	0.87	0.03
123	0.31	0.5	0.67	0.08
7	0.49	0.32	0.72	0.06
1024	0.43	0.33	0.71	0.08
99	0.23	0.53	0.66	0.09

Table 14. Shortcut Isolation for LUSC

seeds	Baseline - error rare		After Exclusion of LUSC - error rate	
	LUAD	LUSC	LUAD	LUSC
42	0.4	0.4	0.02	0.66
123	0.31	0.5	0.06	0.57
7	0.49	0.32	0.03	0.62
1024	0.43	0.33	0.07	0.56
99	0.23	0.53	0.1	0.5

RestNet50V2 Raw Data: 4-Center Setup (Asterand Site)

Table 15. Shortcut Isolation for LUAD

seeds	Baseline - error rare		After Exclusion of LUAD - error rate	
	LUAD	LUSC	LUAD	LUSC
42	0.34	0.39	0.6	0.16
123	0.58	0.15	0.71	0.06
7	0.23	0.57	0.57	0.2
1024	0.75	0.13	0.67	0.08
99	0.63	0.11	0.36	0.29

Table 16. Shortcut Isolation for LUSC

seeds	Baseline - error rare		After Exclusion of LUSC - error rate	
	LUAD	LUSC	LUAD	LUSC
42	0.34	0.39	0.05	0.76
123	0.58	0.15	0.38	0.26
7	0.23	0.57	0.13	0.59
1024	0.75	0.13	0.28	0.35
99	0.63	0.11	0.03	0.72

RestNet50V2 Raw Data: 4-Center Setup (Johns Hopkins Site)

Table 17. Shortcut Isolation for LUAD

seeds	Baseline - error rare		After Exclusion of LUAD - error rate	
	LUAD	LUSC	LUAD	LUSC
42	0.34	0.28	0.49	0.13
123	0.26	0.36	0.41	0.21
7	0.48	0.18	0.68	0.05
1024	0.67	0.06	0.63	0.11
99	0.65	0.08	0.88	0.01

Table 18. Shortcut Isolation for LUSC

seeds	Baseline - error rare		After Exclusion of LUSC - error rate	
	LUAD	LUSC	LUAD	LUSC
42	0.34	0.28	0.21	0.41
123	0.26	0.36	0.2	0.44
7	0.48	0.18	0.11	0.57
1024	0.67	0.06	0.15	0.51
99	0.65	0.08	0.09	0.63

RestNet50V2 Raw Data: 5-Center Setup (Johns Hopkins & University of Pittsburgh)

Table 19. Shortcut Isolation for LUAD

seeds	Baseline - error rare		After Exclusion of LUAD - error rate	
	LUAD	LUSC	LUAD	LUSC
42	0.75	0.19	0.62	0.16
123	0.53	0.24	0.67	0.12
7	0.42	0.36	0.88	0.07
1024	0.54	0.3	0.67	0.15
99	0.43	0.4	0.67	0.11

Table 20. Shortcut Isolation for LUSC

seeds	Baseline - error rare		After Exclusion of LUSC - error rate	
	LUAD	LUSC	LUAD	LUSC
42	0.75	0.19	0.1	0.73
123	0.53	0.24	0.29	0.45
7	0.42	0.36	0.1	0. 7
1024	0.54	0.3	0.18	0.59
99	0.43	0.4	0.06	0.65

DenseNet121 Raw Data: 3-Center Setup (Asterand Site)

Table 21. Shortcut Isolation for LUAD

seeds	Baseline - error rare		After Exclusion of LUAD - error rate	
	LUAD	LUSC	LUAD	LUSC
42	0.44	0.3	0.92	0.004
123	0.64	0.15	0.52	0.18
7	0.53	0.22	0.87	0.012
1024	0.46	0.24	0.58	0.14
99	0.65	0.1	0.80	0.012

Table 22. Shortcut Isolation for LUSC

seeds	Baseline - error rare		After Exclusion of LUSC - error rate	
	LUAD	LUSC	LUAD	LUSC
42	0.44	0.3	0.04	0.65
123	0.64	0.15	0.06	0.64
7	0.53	0.22	0.1	0.52
1024	0.46	0.24	0.21	0.32
99	0.65	0.1	0.03	0.76

DenseNet121 Raw Data: 3-Center Setup (Roswell Park Site)

Table 23. Shortcut Isolation for LUAD

seeds	Baseline - error rare		After Exclusion of LUAD - error rate	
	LUAD	LUSC	LUAD	LUSC
42	0.41	0.28	0.62	0.06
123	0.41	0.29	0.74	0.03
7	0.22	0.48	0.87	0.01
1024	0.3	0.35	0.71	0.04
99	0.51	0.19	0.61	0.07

Table 24. Shortcut Isolation for LUSC

seeds	Baseline - error rare		After Exclusion of LUSC - error rate	
	LUAD	LUSC	LUAD	LUSC
42	0.41	0.28	0.07	0.39
123	0.41	0.29	0.02	0.59
7	0.22	0.48	0.01	0.53
1024	0.3	0.35	0.07	0.39
99	0.51	0.19	0.02	0.54

DenseNet121 Raw Data: 4-Center Setup (Asterand Site)

Table 25. Shortcut Isolation for LUAD

seeds	Baseline - error rare		After Exclusion of LUAD - error rate	
	LUAD	LUSC	LUAD	LUSC
42	0.48	0.18	0.7	0.05
123	0.73	0.03	0.89	0.008
7	0.36	0.35	0.8	0.012
1024	0.64	0.07	0.58	0.11
99	0.52	0.19	0.53	0.14

Table 26. Shortcut Isolation for LUSC

seeds	Baseline - error rare		After Exclusion of LUSC - error rate	
	LUAD	LUSC	LUAD	LUSC
42	0.48	0.18	0.02	0.81
123	0.73	0.03	0.08	0.61
7	0.36	0.35	0.2	0.39
1024	0.64	0.07	0.11	0.49
99	0.52	0.19	0.12	0.47

DenseNet121 Raw Data: 4-Center Setup (Johns Hopkins Site)

Table 27. Shortcut Isolation for LUAD

seeds	Baseline - error rare		After Exclusion of LUAD - error rate	
	LUAD	LUSC	LUAD	LUSC
42	0.56	0.16	0.68	0.06
123	0.42	0.23	0.7	0.05
7	0.54	0.12	0.59	0.08
1024	0.27	0.38	0.7	0.04
99	0.47	0.19	0.5	0.14

Table 28. Shortcut Isolation for LUSC

seeds	Baseline - error rare		After Exclusion of LUSC - error rate	
	LUAD	LUSC	LUAD	LUSC
42	0.56	0.16	0.03	0.78
123	0.42	0.23	0.03	0.81
7	0.54	0.12	0.08	0.63
1024	0.27	0.38	0.13	0.61
99	0.47	0.19	0.07	0.74

DenseNet121 Raw Data: 5-Center Setup (Johns Hopkins & University of Pittsburgh Sites)

Table 29. Shortcut Isolation for LUAD

seeds	Baseline - error rare		After Exclusion of LUAD - error rate	
	LUAD	LUSC	LUAD	LUSC
42	0.67	0.1	0.72	0.04
123	0.53	0.26	0.53	0.06
7	0.51	0.26	0.87	0.01
1024	0.58	0.22	0.67	0.09
99	0.42	0.27	0.93	0.012

Table 30. Shortcut Isolation for LUSC

seeds	Baseline - error rare		After Exclusion of LUSC - error rate	
	LUAD	LUSC	LUAD	LUSC
42	0.67	0.1	0.2	0.1
123	0.53	0.26	0.07	0.72
7	0.51	0.26	0.11	0.6
1024	0.58	0.22	0.12	0.57
99	0.42	0.27	0.05	0.76
