# Supplementary material for: Deceptive bias measurement in deep learning: Assessing shortcut reliance in TCGA cancer models
Source: PLOS Digit Health. 2026 Sep 3;5(9):e0001165. doi: 10.1371/journal.pdig.0001165 (PMC13541255; doi:10.1371/journal.pdig.0001165)
Supplement: Supplementary file 2 [file pdig.0001165.s003.pdf]

Manuscript Number: PDIG-D-25-01189

Article Title: "Deceptive Bias Measurement in Deep Learning: Assessing Shortcut Reliance in TCGA Cancer Models"

Dear Dr. Anthony Celi, [Editor-in-Chief]

Thank you for allowing a resubmission of our manuscript, with an opportunity to address the reviewers' constructive comments to improve the overall quality of the paper. In the following, please find the point-by-point response to the reviewers' comments. All revised parts are marked in the manuscript. Before proceeding to the point-by-point response, a summary of the important revisions applied to the manuscript contributed to major concerns. Hopefully, the revised paper quality would meet all journal's required expectations.

Best Regards,

Farnaz Kheiri

Summary of the important revisions applied to the manuscript:

- The manuscript has been completely restructured to follow standard academic conventions, now including clearly defined sections: Introduction, Related Work, Methodology, Results, Discussion, and Conclusion.

- To ensure the generalizability of the Deceptive Signal, we expanded our experiments to include three distinct architectures: EfficientNetB0, ResNet50V2, and DenseNet121.

- We implemented a multi setting experimental configuration across multiple medical centers and tested varying configurations to analyze bias signals under different domain-shift conditions in 5 separate runs to verify the results validity.

- We added statistical significance testing (including p-values and confidence intervals) to Section 4 to verify the robustness of the observed deceptive signals.

- We have made the complete source code and slide Ids for our implementation publicly available on GitHub.

Comments provided by the reviewers

Reviewer 1

Reviewer Comment: Innovative and clinically relevant paper.

Response: We thank the reviewer for their positive assessment and for recognizing the innovation and clinical relevance of our study.

Reviewer 2

1. **Reviewer Comment:** The main concern is the lack of statistical rigor. The reported results are based on single experimental runs, without repeated training, confidence intervals, or significance testing.

   Response: Thanks for your insightful comment. We fully agree that statistical robustness is essential for validating findings in deep learning, particularly in clinical applications. We have addressed this concern by implementing a comprehensive statistical framework across our entire experiments:

   - **Repeated Experimental Runs:** We have moved beyond single run and now report results based on five independent training cycles (n = 5) using different random seeds for every configuration and architecture.
     The comprehensive results are reported in Table 1-3 on pages 15 and 16.

     The summary of results:
     "The evaluation across Tables 1–3 reveals a consistent failure in generalization across EfficientNetB0, ResNet50V2, and DenseNet121 models when exposed to partial site-specific data. By reporting the mean and standard deviation across five independent runs (utilizing seeds 42, 123, 7, 1024, and 99), these results statistically confirm a deceptive behavioral shift. Specifically, once the model is exposed to partial data from a target external center during training, it demonstrates a tendency to misclassify samples of the excluded class as the included class from that same site. When compared to the baseline established in the full exclusion experiments, this shift quantifies the model's transition from learning morphology to exploiting site-specific shortcuts
     The high magnitude of the Deceptive Signal, which frequently reaches or exceeds 1.0, such as the 1.01 ± 0.11 observed in EfficientNet or the peak of 1.15 ± 0.05 in ResNet, quantifies a heavy reliance on center-specific artifacts rather than morphological features. Ultimately, this cross-architecture synthesis demonstrates that shortcut learning is a fundamental characteristic of these deep learning models, persisting across 3-center, 4-center, and 5-center configurations regardless of the specific network design."

- **Significance Testing:** We performed a one-sample t-test against the null hypothesis for every deceptive signal reported. As shown in Table 4 on page 16, most of our results yielded p-values significantly lower than 0.05, confirming that the detected signals are not the product of stochastic noise.

- **Confidence Intervals and Variance:** All tables (Tables 1–4) have been updated to report the Mean and Standard Deviation. Furthermore, we have included a discussion of 95% Confidence Intervals (95%CI) in Section 4.3 (page 16) to illustrate the precision and reliability of the Deceptive Signal across different runs.

  The summary of results:
  We validated the reliability of the detected deceptive signals using statistical analysis. Each experiment was run with five different random seeds to ensure robustness. Although results varied by model and setup, standard deviations (0.05 – 0.20) stayed within acceptable deep learning ranges. A one-sample t-test against $S = 0$ showed p-values below 0.05 in nearly all cases, confirming statistical significance. Additionally, 95% confidence intervals did not include zero, indicating that the models consistently rely on center-specific shortcuts rather than meaningful task features.

2. **Reviewer Comment:** The experimental validation is limited to one specific setup (two classes, one sensitive center, one architecture). Including additional centers, architectures, or experimental configurations would significantly strengthen the generalizability of the proposed metric.

   Response: Thanks for your comment. In response to this constructive feedback, we have significantly expanded our experimental scope to include the following:

   - **Architectural Diversity:** We moved beyond a single architecture and now provide comprehensive results for three distinct, state-of-the-art models: EfficientNet, ResNet50V2, and DenseNet12 to confirm that the metric consistently detects bias regardless of the underlying model structure.
     The corresponding results for each model have been reported separately in Tables 1–3 on pages 15 and 16. Furthermore, the specific model architectures and training configurations used in this study have been provided in detail in our GitHub repository.

   - **Multiple Sensitive Centers:** We have expanded the experimental design to include a systematic rotation of sensitive data centers. Our analysis now includes rotating evaluations across several distinct medical centers, including Asterand, Roswell Park, Johns Hopkinz, and University of Pittsburgh. In each experimental iteration, a different center was designated as the held out or partially excluded domain.
     We have added Section 3.4.2 (Experimental Setup) on page 10 to provide a detailed discussion of the experimental design applied in this study.

- **Varying Experimental Configurations:** We have expanded the study to include 3-center, 4-center, and 5-center setups to analyze the deceptive shift in the presence of increased levels of domain diversity.
  The specific details of these configurations are provided in Section 3.4.2 on page 10.

  Summary of results:
  Across all expanded experimental settings, our results consistently demonstrate the presence of strong and statistically significant deceptive signals. Specifically, the metric was evaluated under multiple center configurations (3-, 4-, and 5-center setups), across different sensitive center rotations, and using three distinct architectures (EfficientNet-B0, ResNet50V2, and DenseNet121). Despite these variations, all models exhibit the same overall trend: a persistent positive deceptive signal indicating reliance on center-specific shortcuts rather than task-relevant features. While the magnitude of the signal varies slightly across centers and architectures, its consistent presence across all configurations confirms that the proposed metric is robust and generalizes well beyond a single experimental setup

Regarding the focus on two classes, we chose to work with Lung Adenocarcinoma (LUAD) and Lung Squamous Cell Carcinoma (LUSC). These specific types were selected because we already had a significant volume of high-quality, diagnostic-grade patches preprocessed from the previous research studies. By building on this existing foundation, we could ensure much higher data integrity to avoid the technical artifacts often found in less reliable slides (such as frozen sections).
The clarification regarding this point has been added to the first paragraph of Section 3.4.1 on page 9.

3. **Reviewer Comment:** Reproducibility also needs improvement. While TCGA is publicly available, the manuscript does not clearly specify the exact slide IDs, train/test splits, or provide code for the Deceptive Pipeline.

   Response: Thanks for pointing this out. To address these concerns, we have established a public GitHub repository "https://github.com/FarnazKheiri/Deceptive-Bias-Measurement-in-Deep-Learning" that includes the specific TCGA slide IDs, and the complete source code for the Deceptive Pipeline. Furthermore, we have provided a Supplementary File alongside the manuscript containing the raw numerical results from each independent experimental run, which provides full visibility into the individual data points behind the reported means and standard deviations.

4. **Reviewer Comment:** The manuscript is generally understandable but would benefit from careful language editing to correct recurring grammatical and typographical errors.

Response: We appreciate your feedback regarding the presentation of the manuscript. In response, the entire text has been thoroughly revised and edited to correct grammatical and typographical errors.

Reviewer 3

1. **Reviewer Comment:** The metric has been demonstrated on histopathology images only. It would be great if it were also validated on other types of data such as text or natural images.

   Response: We appreciate your valuable suggestion. In this work, we intentionally focus on histopathology and the TCGA dataset, as medical imaging represents a high-stakes domain where shortcut learning can directly impact clinical decision-making. Our goal was to first provide a controlled and in-depth validation of the proposed Deceptive Pipeline within this specific and clinically relevant setting. While extending the framework to other modalities such as natural images or text is certainly an interesting and important direction, it requires a fundamental redesign of the experimental pipeline due to the inherently different nature of these data types, including differences in feature structure, representation, and sources of bias. Such an extension is therefore beyond the scope of the current study and would require a separate and extensive set of experiments tailored to each modality. Nonetheless, we fully agree that cross-modal validation is a valuable future direction, and we are actively exploring the application of the proposed framework to additional data domains, including text, in future work.

2. **Reviewer Comment:** It is also often argued that a thorough Computer Vision bias audit typically combines subgroup performance metrics, dataset representation analysis, saliency auditing, and evaluation on dedicated fairness benchmarks rather than relying on any single metric. How do you address this in this study?

   Response: We completely agree that bias is a multifaceted problem and cannot be fully captured by a single metric. Our proposed Deceptive Signal is therefore not intended to replace established bias auditing techniques such as subgroup performance analysis, saliency maps, or fairness benchmark evaluations, but rather to complement them by providing a complementary quantitative perspective. While existing methods are highly effective at identifying performance disparities across groups or offering qualitative interpretability insights, they often do not explicitly quantify the extent to which a model relies on shortcut mechanisms such as site-specific or spurious correlations. In contrast, the Deceptive Signal provides a standardized, behavior-based numerical measure that captures these shortcut dynamics directly by evaluating performance shifts under controlled partial information settings. This makes it particularly useful for diagnosing how bias is exploited by the model, rather than only indicating that bias exists. We believe this additional perspective offers significant value when combined with existing fairness auditing tools, enabling a more complete understanding of model bias.

   We have added a clarification in the paragraph 3 of Section 2.2 (Related Work) on page 4.

3. **Reviewer Comment:** The paper needs to be structured with proper sections such as Introduction, Related Work, Methodology, Results, Discussion and Conclusion.

Response: Thanks for your valuable comment. We have completely restructured the paper to align with standard academic conventions and the specific sections requested.

- Section 1: Introduction
- Section 2: Related Work (categorizing prior bias detection and fairness evaluation methods).
- Section 3: Methodology (including The Deceptive Pipeline Principle, Mathematical Formalization, Probabilistic Method Justification and Description of Case Study).
- Section 4: Results (featuring the Experimental Procedure on TCGA, comprehensive performance tables and Statistical Significance Analysis).
- Section 5: Discussion
- Section 6: Conclusion (summarizing findings, limitations, and future work).

4. **Reviewer Comment:** The Introduction lacks specific research questions that are guiding the research work.

   Response: Thanks for your insightful comment. We have revised the Introduction to include three primary research questions guiding this study:

   - RQ1 addresses the extent of site-specific artifact reliance in multi-center datasets.
   - RQ2 focuses on the effectiveness of the controlled exclusion-inclusion training regime for isolating shortcuts.
   - RQ3 evaluates the stability and architecture-agnostic nature of the proposed Deceptive Signal.

   These questions are now presented on the last paragraph of page 2.

   To ensure these questions are not only stated but also empirically grounded, all conducted experiments are explicitly designed to address them: the multi-center and rotation settings directly evaluate RQ1, the partial inclusion/exclusion training protocol operationalizes RQ2 by inducing and measuring shortcut reliance, and the cross-architecture experiments together with statistical significance analysis support RQ3 by demonstrating consistent behavior across different model backbones and configurations.

5. **Reviewer Comment:** What is Author Summary? Is a summary of what the paper is all about or a summary of the background of the authors?

   Response: Thanks for your valuable comment. The "Author Summary" is a mandatory requirement for PLOS Digital Health. Unlike a traditional abstract, its purpose is to provide a non-technical, first-person summary of the research for a non-specialist audience, highlighting the work's significance and real-world implications.

6. **Reviewer Comment:** Labelling of figures also needs improvement - e.g. Fig 3. This figure represents the overview of the partial Shortcut exclusion Scenario... should have been Fig 3. An overview of the partial Shortcut exclusion Scenario...

   Response: We appreciate your feedback. We have checked all figure captions throughout the manuscript to be more direct and concise.

7. **Reviewer Comment:** Some inverted commas are pointing in the wrong direction e.g. in ”cheating.”

   Response: We thank the reviewer for identifying this typographical error. We have corrected the orientation of all quotation marks throughout the revised manuscript.
