# Supplementary material for: Deceptive bias measurement in deep learning: Assessing shortcut reliance in TCGA cancer models
Source: PLOS Digit Health. 2026 Sep 3;5(9):e0001165. doi: 10.1371/journal.pdig.0001165 (PMC13541255; doi:10.1371/journal.pdig.0001165)
Supplement: Supplementary file 3 [file pdig.0001165.s004.pdf]

Manuscript Number: PDIG-D-25-01189

Article Title: "Deceptive Bias Measurement in Deep Learning: Assessing Shortcut Reliance in TCGA Cancer Models"

Dear Editors,

Thank you for giving us the opportunity to submit the second revised version of our manuscript. We sincerely appreciate your time and the reviewers' constructive comments.

In this revision, we have carefully addressed the remaining minor issues, including language and typographical corrections, sentence-level revisions, and refinement of statements related to generalizability and model trustworthiness. The revised parts have been marked in the manuscript for easier review.

We hope that the revised manuscript now meets the journal's expectations and is suitable for publication.

Best regards,
Farnaz Kheiri

Comments provided by the reviewers

# Reviewer 1

Thank you for your positive comment and for recognizing the clinical relevance of our work.

# Reviewer 2

Reviewer Comment: Only minor issues remain. The authors should correct a few remaining language and typographical errors and slightly temper broad claims about generalizability and model trustworthiness. These points do not prevent publication.

Response: Thank you for your constructive comments.

The manuscript has been carefully reviewed and professionally proofread. Typographical errors, grammatical issues, and minor language inconsistencies have been corrected throughout the revised manuscript.

Also. we carefully reviewed the manuscript and revised statements that could be interpreted as overly broad claims regarding the model's generalizability and trustworthiness. To illustrate these revisions, we have listed several representative examples below.

1. Abstract

Original sentence:

"… provides a concrete value representing the model's susceptibility."

Revised sentence:

"… provides a quantitative estimate of the model's susceptibility."

2. Abstract

Original sentence:

"… successfully quantified strong dependencies …"

Revised sentence:

"… provides a quantitative estimate of the model's susceptibility …"

3. Author summary, Paragraph 1:

Original sentence:

"… AI systems appear accurate at first glance <u>while remaining unreliable</u> for real-world patient care settings."

Revised sentence:

"… AI systems appear accurate at first glance while <u>potentially limiting reliability</u> in real-world patient care settings."

3. Author summary, Paragraph 2:

Original sentence:

"… we calculate a <u>precise score</u>."

Revised sentence:

"… calculate a <u>quantitative score</u>."

3. Author summary, Paragraph 2:

Original sentence:

"… thereby ensuring that medical decisions are driven by biology, not background noise."

Revised sentence:

"… helping assess whether predictions are more strongly driven by task-relevant biological features rather than background artifacts."

Reviewer 3

Thank you for your positive comment. We are pleased that the previous concerns have been adequately addressed.
