# Supplementary material for: Deceptive bias measurement in deep learning: Assessing shortcut reliance in TCGA cancer models
Source: PLOS Digit Health. 2026 Sep 3;5(9):e0001165. doi: 10.1371/journal.pdig.0001165 (PMC13541255; doi:10.1371/journal.pdig.0001165)
Supplement: Peer review history

# Peer review: Decision Letter 0

**DOI:** 10.1371/journal.pdig.0001165.r001

**Authors:** Jing Tian, Po-Chih Kuo

12 Mar 2026

Response to ReviewersRevised Manuscript with Track ChangesManuscript **Journal Requirements:**

1. We ask that a manuscript source file is provided at Revision. Please upload your manuscript file as a .doc, .docx, .rtf or .tex.

**Additional Editor Comments (if provided):**  **Reviewers' Comments:**

**Comments to the Author**

1. Does this manuscript meet PLOS Digital Health’s publication criteria?

Reviewer #1: Yes

Reviewer #2: No

Reviewer #3: Yes

**********

2. Has the statistical analysis been performed appropriately and rigorously?

Reviewer #1: Yes

Reviewer #2: No

Reviewer #3: Yes

**********

3. Have the authors made all data underlying the findings in their manuscript fully available (please refer to the Data Availability Statement at the start of the manuscript PDF file)?

The PLOS Data policy

Reviewer #1: Yes

Reviewer #2: No

Reviewer #3: Yes

**********

4. Is the manuscript presented in an intelligible fashion and written in standard English?

Reviewer #1: Yes

Reviewer #2: No

Reviewer #3: Yes

**********

Reviewer #1: Innovative and clinically relevant paper. Innovative and clinically relevant paper. Innovative and clinically relevant paper. Innovative and clinically relevant paper. Innovative and clinically relevant paper.

Reviewer #2: This manuscript addresses an important and timely issue in medical AI: shortcut learning in multicenter datasets. The proposed Deceptive Signal metric is conceptually interesting, clearly motivated, and mathematically well structured. The TCGA case study is relevant and helps illustrate the core idea.

However, in its current form, the work requires methodological strengthening before the conclusions can be fully supported.

The main concern is the lack of statistical rigor. The reported results are based on single experimental runs, without repeated training, confidence intervals, or significance testing. Given the stochastic nature of deep learning, it is essential to demonstrate that the observed effects are stable and reproducible rather than due to random variation.

Additionally, the experimental validation is limited to one specific setup (two classes, one sensitive center, one architecture). Including additional centers, architectures, or experimental configurations would significantly strengthen the generalizability of the proposed metric.

Reproducibility also needs improvement. While TCGA is publicly available, the manuscript does not clearly specify the exact slide IDs, train/test splits, or provide code for the Deceptive Pipeline.

Finally, the manuscript is generally understandable but would benefit from careful language editing to correct recurring grammatical and typographical errors.

In summary, the idea is promising and relevant to digital health, but the study requires stronger statistical validation, broader experimental support, and improved presentation before it can be considered for publication.

Reviewer #3: What are the main claims of the paper and how significant are they for the discipline?

The paper proposes the Deceptive Signal metric, a novel quantitative measure designed to assess the extent of a deep learning model’s reliance on hidden shortcuts during the learning process, whereby models exploit signals linked to sensitive attributes, such as data source or collection site, instead of relying on task, relevant features. Although standard metrics such as accuracy, F1 score, etc, can look good, a model can be systematically wrong for specific subgroups (hence deceptive bias) as the bias hides behind aggregate performance numbers.

Deceptive bias, in particular, is one of the core concerns in AI ethics because it can cause real-world harm while remaining invisible to standard quality checks.

Are these claims novel? If not, please specify papers that weaken the claims of originality of this one. If a similar paper was recently published, the current manuscript may still be eligible for publication under our complementary research policy?

The claim is novel as it moves beyond simple accuracy checks by introducing a specific quantitative metric for shortcut learning, through a testing framework that forces a model into controlled training scenarios, deliberately withholding specific "shortcut" information to see how the model reacts. By mathematically comparing the model’s behavior across these scenarios, the authors calculate a precise score that indicates the magnitude of the model’s dependence on irrelevant patterns. This metric allows to put a concrete number on a model’s trustworthiness and ensuring that medical decisions are driven by biology, not background noise.

Are the claims properly placed in the context of the previous literature? Have the authors treated the literature fairly?

The claim has been properly contextualized in literature. Many existing bias investigation methods tend to assess bias after the model is trained, by measuring how its predictions vary across subgroups. However, such outcome-based evaluations may fail to detect whether the model has relied on task-irrelevant features embedded within specific subgroups. To truly ensure fairness and generalization, it is crucial to go beyond surface-level performance metrics and analyze the training process itself by examining whether the model is learning representations that generalize to the task or simply memorizing subgroup-specific artifacts.

Do the data and analyses fully support the claims? If not, what other evidence is required?

The authors used the TCGA histopathology dataset to demonstrate the feasibility of their work.

Would additional work improve the paper? How much better would the paper be if this work were performed and how difficult would it be to do this work?

The metric has been demonstrated on histopathology images only. It would be great if it were also validated on other types of data such as text or natural images.

It is also often argued that a thorough Computer Vision bias audit typically combines subgroup performance metrics, dataset representation analysis, saliency auditing, and evaluation on dedicated fairness benchmarks rather than relying on any single metric. How do you address this in this study?

If a protocol is provided, for example for a randomized controlled trial, are there any important deviations from it? If so, have the authors explained adequately why the deviations occurred?

No

PLOS Digital Health encourages authors to publish detailed protocols and algorithms as supporting information online. Do any particular methods used in the manuscript warrant such treatment?

No

If the paper is considered unsuitable for publication in its present form, does the study itself show sufficient potential that the authors should be encouraged to resubmit a revised version?

N/A

Are original data deposited in appropriate repositories and accession/version numbers provided for genes, proteins, mutants, diseases, etc.?

Secondary data used - https://portal.gdc.cancer.gov

Does the study conform to any relevant guidelines such as CONSORT, MIAME, QUORUM, STROBE, and the Fort Lauderdale agreement?

N/A

Are details of the methodology sufficient to allow the experiments to be reproduced?

Yes.

Is any software created by the authors freely available?

N/A

Is the manuscript well organized and written clearly enough to be accessible to non-specialists?

It needs improvement:

- The paper needs to be structured with proper sections such as Introduction, Related Work, Methodology, Results, Discussion and Conclusion. This will help a reader to follow the paper easily.

- The Introduction lacks specific research questions that are guiding the research work.

- What is Author Summary? Is a summary of what the paper is all about or a summary of the background of the authors?

- Labelling of figures also needs improvement - e.g. Fig 3. This figure represents the overview of the partial Shortcut exclusion Scenario... should have been Fig 3. An overview of the partial Shortcut exclusion Scenario...

- Some inverted commas are pointing in the wrong direction e.g. in ”cheating.”

**********

what does this mean?). If published, this will include your full peer review and any attached files.

**Do you want your identity to be public for this peer review?**   If you choose “no”, your identity will remain anonymous but your review may still be made public.

For information about this choice, including consent withdrawal, please see our Privacy Policy

Reviewer #1: No

Reviewer #2: No

Reviewer #3: No

**********

**Figure resubmission:**

**Reproducibility:**

To enhance the reproducibility of your results, we recommend that authors of applicable studies deposit laboratory protocols in protocols.io, where a protocol can be assigned its own identifier (DOI) such that it can be cited independently in the future. Additionally, PLOS ONE offers an option to publish peer-reviewed clinical study protocols. Read more information on sharing protocols at https://plos.org/protocols?utm_medium=editorial-email&utm_source=authorletters&utm_campaign=protocols

# Author comment: Author response to Decision Letter 1

**DOI:** 10.1371/journal.pdig.0001165.r002

10 May 2026

Attachment Submitted filename: Response to Reviewers.pdf

# Peer review: Decision Letter 1

**DOI:** 10.1371/journal.pdig.0001165.r003

**Authors:** Jing Tian, Po-Chih Kuo

1 Jun 2026

Response to ReviewersRevised Manuscript with Track ChangesManuscript **Journal Requirements:**

1. Please upload separate figure files in .tif or .eps format. Also, remove the figures from your manuscript file but keep the legends.

For more information about figure files please see our guidelines:

https://journals.plos.org/digitalhealth/s/figures

https://journals.plos.org/digitalhealth/s/figures#loc-file-requirements

2. We have noticed that you have uploaded Supporting Information files, but you have not included a list of legends. Please add a full list of legends for your Supporting Information files after the references list.

**Additional Editor Comments (if provided):**  **Reviewers' Comments:**

**Comments to the Author**

Reviewer #1: All comments have been addressed

Reviewer #2: All comments have been addressed

Reviewer #3: All comments have been addressed

**********

publication criteria?

Reviewer #1: Yes

Reviewer #2: Partly

Reviewer #3: Yes

**********

3. Has the statistical analysis been performed appropriately and rigorously?

Reviewer #1: Yes

Reviewer #2: Yes

Reviewer #3: Yes

**********

4. Have the authors made all data underlying the findings in their manuscript fully available (please refer to the Data Availability Statement at the start of the manuscript PDF file)?

The PLOS Data policy

Reviewer #1: Yes

Reviewer #2: Yes

Reviewer #3: Yes

**********

5. Is the manuscript presented in an intelligible fashion and written in standard English?

Reviewer #1: Yes

Reviewer #2: Yes

Reviewer #3: Yes

**********

**Reviewer #1:**   Highly clinically relevant paper.

**Reviewer #2:**  The revised manuscript is much improved and, in my view, is now suitable for publication after minor editorial adjustments.

The authors have addressed the main concerns from the previous review. The manuscript is better structured, the research questions are clearer, and the experimental design is stronger. The addition of multiple architectures, different center configurations, repeated runs with random seeds, and statistical testing makes the work more technically convincing.

The proposed Deceptive Signal metric addresses an important problem in medical AI: shortcut learning and reliance on site-specific artifacts. The study is limited to TCGA histopathology images and two cancer classes, but the authors now present the work more clearly as a focused methodological contribution rather than a definitive clinical validation.

Data and code availability have also improved, with TCGA identified as the source dataset and a GitHub repository provided with slide IDs and code.

Only minor issues remain. The authors should correct a few remaining language and typographical errors and slightly temper broad claims about generalizability and model trustworthiness. These points do not prevent publication.

Overall, I support publication after minor revision.

**Reviewer #3:**   All previous concerns have been adequately addressed.

**********

what does this mean?). If published, this will include your full peer review and any attached files.

**Do you want your identity to be public for this peer review?**   If you choose “no”, your identity will remain anonymous but your review may still be made public.

For information about this choice, including consent withdrawal, please see our Privacy Policy

Reviewer #1: No

Reviewer #2: No

Reviewer #3: **Yes:**  Bennett Kankuzi

**********

**Figure resubmission:**

**Reproducibility:**

To enhance the reproducibility of your results, we recommend that authors of applicable studies deposit laboratory protocols in protocols.io, where a protocol can be assigned its own identifier (DOI) such that it can be cited independently in the future. Additionally, PLOS ONE offers an option to publish peer-reviewed clinical study protocols. Read more information on sharing protocols at https://plos.org/protocols?utm_medium=editorial-email&utm_source=authorletters&utm_campaign=protocols

# Author comment: Author response to Decision Letter 2

**DOI:** 10.1371/journal.pdig.0001165.r004

29 Jun 2026

Attachment Submitted filename: Response_to_Reviewers_auresp_2.pdf

# Editor report: Decision Letter 2

**DOI:** 10.1371/journal.pdig.0001165.r005

**Authors:** Jing Tian, Po-Chih Kuo

2 Aug 2026

Deceptive Bias Measurement in Deep Learning: Assessing Shortcut Reliance in TCGA Cancer Models

PDIG-D-25-01189R2

Dear AI researcher Kheiri,

We are pleased to inform you that your manuscript 'Deceptive Bias Measurement in Deep Learning: Assessing Shortcut Reliance in TCGA Cancer Models' has been provisionally accepted for publication in PLOS Digital Health.

Before your manuscript can be formally accepted you will need to complete some formatting changes, which you will receive in a follow-up email from a member of our team.

Please note that your manuscript will not be scheduled for publication until you have made the required changes, so a swift response is appreciated.

IMPORTANT: The editorial review process is now complete. PLOS will only permit corrections to spelling, formatting or significant scientific errors from this point onwards. Requests for major changes, or any which affect the scientific understanding of your work, will cause delays to the publication date of your manuscript.

If your institution or institutions have a press office, please notify them about your upcoming paper to help maximize its impact. If they'll be preparing press materials, please inform our press team as soon as possible -- no later than 48 hours after receiving the formal acceptance. Your manuscript will remain under strict press embargo until 2 pm Eastern Time on the date of publication. For more information, please contact digitalhealth@plos.org.

Thank you again for supporting Open Access publishing; we are looking forward to publishing your work in PLOS Digital Health.

Best regards,

Jing Tian, PhD

Academic Editor

PLOS Digital Health

***********************************************************

**Additional Editor Comments (if provided):**

I checked the revised manuscript and the authors’ response letter and agree that the authors have addressed the minor concerns raised by Reviewer 2.

**Reviewer Comments (if any, and for reference):**
